# Absolute Configuration of 12*S*-Deoxynortryptoquivaline from Ascidian-Derived Fungus *Aspergillus clavatus* Determined by Anisotropic NMR and Chiroptical Spectroscopy

**DOI:** 10.1021/acs.jnatprod.3c01157

**Published:** 2024-01-30

**Authors:** Elisa Doro-Goldsmith, Qi Song, Xiao-Lu Li, Xiao-Ming Li, Xue-Yi Hu, Hong-Lei Li, Hao-Ran Liu, Bin-Gui Wang, Han Sun

**Affiliations:** † Leibniz-Forschungsinstitut für Molekulare Pharmakologie (FMP), Robert-Rössle-Strasse 10, Berlin 13125, Germany; ‡ School of Chemistry, The University of Edinburgh, David Brewster Road, Edinburgh EH9 3FJ, United Kingdom; § CAS and Shandong Province Key Laboratory of Experimental Marine Biology, Institute of Oceanology, Chinese Academy of Sciences, Nanhai Road 7, Qingdao 266071, China; ∥ Institute of Chemistry, Technische Universität Berlin, Straße des 17. Juni 135, Berlin 10623, Germany; ⊥ University of Chinese Academy of Sciences, Yuquan Road 19A, Beijing 100049, China

## Abstract

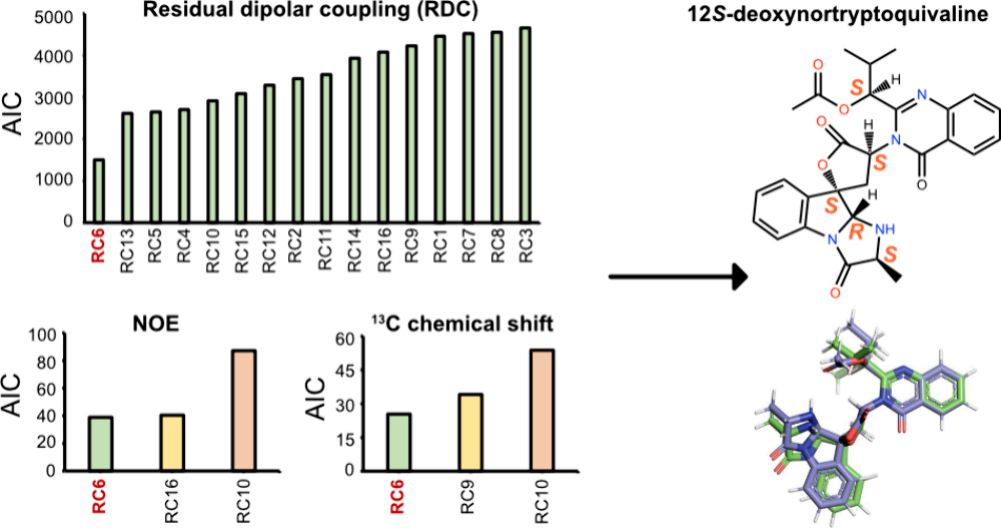

Tryptoquivalines are highly toxic metabolites initially isolated from the fungus *Aspergillus clavatus*. The relative and absolute configuration of tryptoquivaline derivates was primarily established by comparison of the chemical shifts, NOE data, and ECD calculations. A de novo determination of the complete relative configuration using NMR spectroscopy was challenging due to multiple spatially separated stereocenters, including one nonprotonated carbon. In this study, we isolated a new tryptoquivaline derivative, 12*S*-deoxynortryptoquivaline (**1**), from the marine ascidian-derived fungus *Aspergillus clavatus* AS-107. The correct assignment of the relative configuration of **1** was accomplished using anisotropic NMR spectroscopy, while the absolute configuration was determined by comparing calculated and experimental ECD spectra. This case study highlights the effectiveness of anisotropic NMR parameters over isotropic NMR parameters in determining the relative configuration of complex natural products without the need for crystallization.

The tryptoquivalines constitute a series of highly toxic metabolites that induce tremors and were initially discovered in *Aspergillus clavatus* in 1975.^1^ This fungus was one of several collected from mold-infested rice found in a Thai household where a child had died from an unidentified toxicosis. The complete relative and absolute configurations of the tryptoquivalines remained unknown until 1979, when nortryptoquivaline was revealed by Springer using single-crystal X-ray diffraction.^2^ Tryptoquivaline derivatives have also been isolated from other fungi, such as the mangrove-derived fungus *Cladosporium* sp. PJX-41.^3^ These tryptoquivalines have shown significant activity against influenza virus A (H1N1). Furthermore, subsequent in silico studies have suggested deoxytryptoquivaline and deoxynortryptoquivaline as potential inhibitors to the SARS-CoV-2 main protease (Mpro),^4,5^ which is currently considered as a key target for preventing infectious diseases caused by the SARS-CoV-2 virus.^6^

Over the past two decades, anisotropic NMR spectroscopy has revolutionized the field of structural elucidation of complex natural products, eliminating the need for crystallization.^7−9^ Anisotropic NMR parameters, such as residual dipolar coupling (RDC), residual chemical shift anisotropy (RCSA), and residual quadrupolar coupling (RQC), have been used extensively to determine the constitution, relative configuration, and conformation of challenging organic molecules.^10−15^ These long-range NMR parameters prove particularly valuable in studies involving molecules with multiple spatially separated stereocenters, proton-deficient molecules, and conformationally flexible molecules.

In this study, we characterized a new tryptoquivaline alkaloid, 12*S*-deoxynortryptoquivaline (**1**), from the marine ascidian-derived fungus *Aspergillus clavatus* AS-107. Here, we employed anisotropic NMR spectroscopy to determine the relative configuration of compound **1** (Figure 1). 12*S*-Deoxynortryptoquivaline presents a substantial challenge for conventional stereochemical elucidation using NMR spectroscopy. This is because the molecule features five stereocenters, some of which are spatially separated and one of which is a quaternary carbon. To address this challenge, we adopted a cross-validation approach, leveraging the recent development of the program *StereoFitter*. *StereoFitter* is a module in Mnova from Mestrelab Research that can incorporate various types of isotropic and anisotropic NMR parameters for stereochemical and conformational analysis. Notably, in this study, the analysis of RDCs alone effectively distinguished the correct relative configuration among the 16 possible ones. Although isotropic NMR data, including ^13^C chemical shifts and NOEs, supported the results obtained from the RDC analysis, they did not offer the same level of discrimination as the anisotropic NMR analysis. Finally, we conclusively determined the absolute configuration of 12*S*-deoxynortryptoquivaline (**1**) by comparing the computed and experimental ECD spectra.

**Figure 1:**
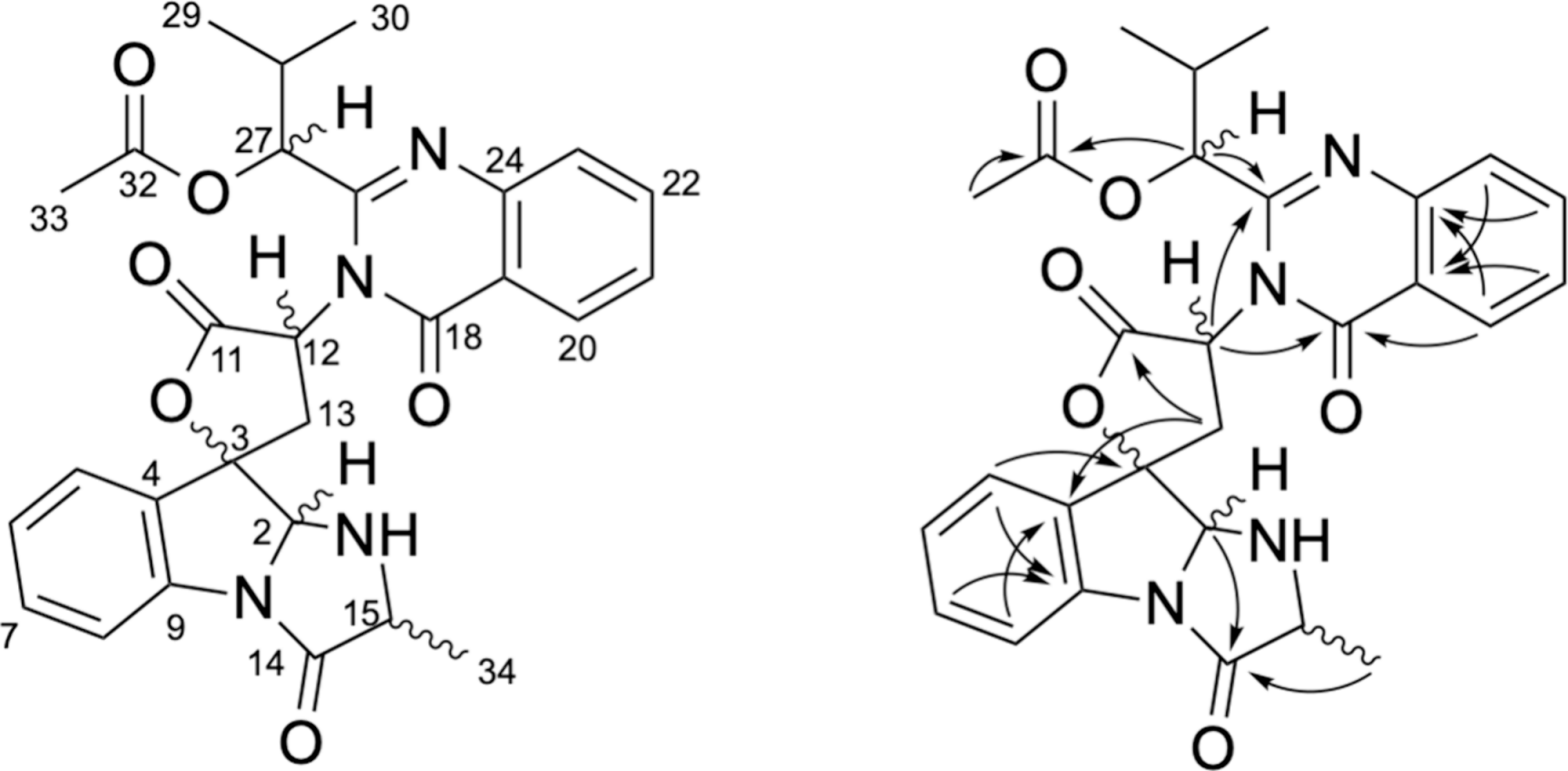
Chemical structure and key HMBC correlations (indicated as arrows) of compound **1**.

## Results and Discussion

### Identification of 12*S*-Deoxynortryptoquivaline (**1**)

Compound **1** was initially isolated as a white amorphous powder. Its molecular formula was determined to be C_28_H_28_O_6_N_4_ by analysis of HRESIMS data at *m*/*z* 517.2079 [M + H]^+^ (calculated for C_28_H_29_O_6_N_4_, 517.2082, Figure S1), requiring 17 degrees of unsaturation. The ^1^H and ^13^C chemical shift assignments of the compound were performed using 1D ^1^H and ^13^C, and 2D COSY, HSQC, and HMBC spectra (Table 1, Figures S2–S7). From the HSQC spectrum, eight individual chemical shifts were identified within an overlapped aromatic portion of the ^1^H spectrum (δ_H_ = 7.37–8.24 ppm). The COSY and HMBC spectra were used to distinguish the two aromatic ring systems at C4–C9 and C19–C24 and to assign the ^1^H and ^13^C signals. Quaternary carbons were identified by comparing ^13^C and HSQC spectra, and their assignments were achieved using the HMBC spectrum, with key correlations shown in Figure 1. All ^1^H and ^13^C chemical shifts of compound **1** could be confidently assigned, aside from prochiral C13–H13a/b methylene and prochiral methyl groups C29–H29 and C30–H30, which could not be distinguished with certainty. The ^1^H and ^13^C NMR data of compound **1** are similar to those of deoxynortryptoquivaline^3^ isolated from the mangrove-derived fungus *Cladosporium* sp. PJX-41. Further structural identification confirmed that they should have the same planar structure, but different relative and absolute configurations.

**Table 1:** NMR Spectroscopic Data (750 MHz, CH_3_OH-*d*_4_) for 12*S*-Deoxynortryptoquivaline (**1**)

position	δ_C_, type	δ_H_ (*J* in Hz)
2	87.1, CH	5.48, s
3	87.8, C	
4	133.3, C	
5	126.8, CH	7.78, d (8.2)
6	127.5, CH	7.37, t (7.6)
7	132.8, CH	7.55, m
8	118.4, CH	7.52, m
9	142.2, C	
11	172.5, C	
12	56.9, CH	5.93, t (9.8)
13	32.6, CH_2_	2.77, dd (13.1, 9.4)
		3.54, dd (13.1, 10.2)
14	179.2, C	
15	61.6, CH	3.83, q (7.0)
18	163.1, C	
19	121.8, C	
20	127.5, CH	8.23, d (8.0)
21	129.2, CH	7.60, t (7.7)
22	136.5, CH	7.89, t (7.7)
23	128.5, CH	7.76, d (8.5)
24	147.7, C	
26	154.8, C	
27	81.0, CH	5.64, d (9.7)
28	33.5, CH	2.49, m
29 or 30	19.1, CH_3_	0.97, d (6.5)
29 or 30	19.4, CH_3_	1.17, d (6.7)
32	171.8, C	
33	20.7, CH_3_	2.22, s
34	17.9, CH_3_	1.50, d (7.0)

### Measurement of NMR Structural Data

The ^3^*J*_HH_ couplings of compound **1** were measured from the isotropic ^1^H spectrum (Table 1). NOE correlations were measured from the ^1^H–^1^H-NOESY spectrum, and peak intensities were extracted from the correlations to calculate ^1^H–^1^H distances according to the isolated spin-pair approximation (Table S1). As part of this calculation, a reference distance of 1.78 Å was used between geminal protons H13a and H13b.

To measure the RDCs of **1**, we used an oligopeptide-based alignment medium, AAKLVFF (28.8 mg/mL), in MeOH-*d*_4_ (Table S2).^17^ In methanol, AAKLVFF undergoes a self-assembly process to form nanotubes over several days. During this time, the degree of alignment of the analyte increases, and the necessary spectra can be acquired using standard NMR equipment and experiments. For RDC extraction, we acquired two ^1^H–^13^C-CLIP-HSQC spectra^16^ under the isotropic and fully aligned, anisotropic states (Figure S8). This anisotropic state was achieved 4 days after AAKLVFF was added to the sample, resulting in a deuterium splitting of 13.5 Hz (Figure S9). The RDCs were calculated as the difference between couplings in the fully aligned state and the isotropic state. In total, 18 ^1^*D*_CH_ RDCs were measured for compound **1**, with only one coupling (C13–H13) not being measured due to an irregular peak shape in the anisotropic spectrum (Table S2).

Additionally, we acquired ΔΔRCSAs of compound **1** from the same sample following the previously proposed procedure.^17^ These ΔΔRCSA values were extracted from two anisotropic ^13^C spectra measured on days 1 and 4 using three references: (i) C-28, which exhibited the smallest theoretically calculated chemical shift anisotropy (CSA); (ii) C-12, which exhibited the second-smallest theoretically calculated CSA; and (iii) the MeOH-*d*_4_ solvent. In total, we measured 26 ΔΔRCSAs for compound **1** (Table S3).

### Determination of the Relative Configuration

Compound **1** possesses five unknown stereocenters at C2, C3, C12, C15, and C27 (Figure 1), resulting in 16 possible relative configurations, RC1–RC16 (Table S4). To determine the correct relative configuration, we employed a cross-validation approach. For this, we first generated a conformational ensemble for each possible relative configuration through a combination of a molecular mechanics (MM)-based conformational search and density functional theory (DFT)-based structural optimizations. As a result, we obtained a structural ensemble ranging from 3 to 19 conformers for each relative configuration (Table S4). The detailed procedure is described in the Computational Methods.

**Figure 2:**
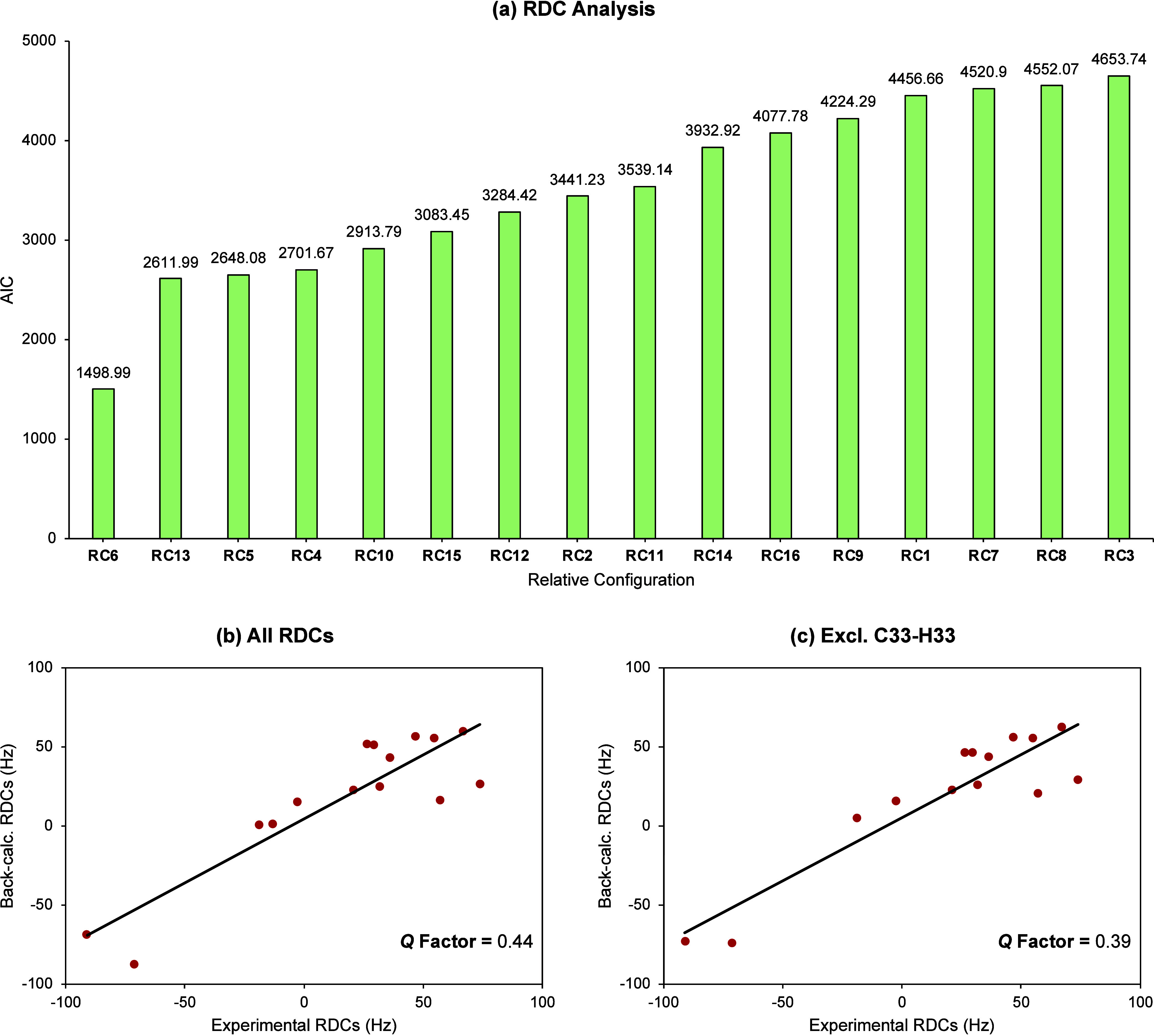
(a) Comparison of the AIC value derived from the RDC analysis for the 16 possible relative configurations, and the correlation between back-calculated and experimental RDCs using (b) all 15 ^1^*D*_CH_ values and (c) excluding the RDC value of C33–H33, which is located on a flexible side-chain.

We then performed a fitting of the 16 obtained structural ensembles against 15 ^1^*D*_CH_ values. This fitting included all couplings except for prochiral C13–H13a/b, which were omitted due to their relatively large experimental errors, and the prochiral methyls C29–H29 and C30–H30, as these could not be confidently distinguished. In this analysis, a single alignment tensor approximation was considered, where all conformers adopt the same alignment tensor.^18^ The calculations were carried out using the program package *StereoFitter*, a recent MNova plug-in for the structural elucidation of organic molecules based on isotropic and anisotropic NMR data. Through *StereoFitter*, the alignment tensor and the conformational population were determined based on a model selection procedure that employed a combination of the Levenberg–Marquardt least-squares optimization and the Akaike Information Criterion (AIC).^19,20^ The determined conformer population and tensor parameters were subsequently used to back-calculate the theoretical RDCs, and the agreement between these predicted and experimental values was assessed through the AIC value. A decrease in the AIC indicates an improved accuracy of the selected model, suggesting that the configuration with the smallest AIC is the most likely relative configuration. The results summarized in Figure 2 clearly demonstrate that the RC6 configuration (C2:R*, C3:S*, C12:S*, C15:S*, C27:S*) exhibits the best agreement between the back-calculated and experimental RDC data. The second-ranking configuration showed a nearly doubled AIC value as compared to the best-ranking one, indicating a very high discrimination probability of the RDC data.

Furthermore, we plotted the back-calculated RDCs against the experimental ones and calculated the *Q* factor^21^ for the top-ranked configuration, RC6. It is noteworthy that incorporating all 15 RDCs during the fitting process resulted in only a moderate level of correlation, yielding a *Q* factor of 0.44 (Figure 2b). Two primary factors could potentially account for the intermediate correlation observed between the experimental and back-calculated RDC data: (i) Employing AAKLVFF as the alignment medium, we obtained very large RDC values for compound **1**, ranging from −91 to 74 Hz. Generally, an optimal alignment should yield RDCs within the range of 10–30 Hz. Values substantially exceeding this range introduce second-order effects in the NMR spectrum, causing signal distortion and subsequently leading to larger errors in RDC measurements. Achieving an optimal RDC range often involves multiple optimization steps;^22^ however, given the limited availability of novel natural products such as compound **1**, conducting an extensive optimization procedure was not feasible. (ii) The initial ensembles generated through an MM-based conformational search and subsequent DFT optimizations, followed by conformer filtering based on the DFT-derived energy, did not adequately account for conformational flexibility. For instance, when excluding the RDC value corresponding to C33–H33, situated in a flexible side-chain, a reduced *Q* factor of 0.39 was obtained (Figure 2b). Nevertheless, despite the correlation and *Q* factor for the correct relative configuration RC6 being suboptimal, the differentiation of the best-fitting configuration from the others was sufficient to safely assign the relative configuration in this scenario.

**Figure 3:**
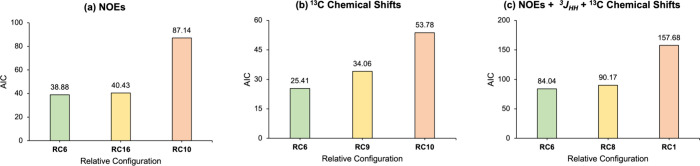
Comparison of the AIC values for the lowest (green), second-lowest (yellow), and highest (red) scoring relative configurations when using (a) NOEs, (b) ^13^C chemical shifts, and (c) a combination of the two along with ^3^*J*_HH_ couplings.

In addition, we employed isotropic structural NMR data, including NOEs (Figure 3a) and ^13^C chemical shifts (Figure 3b), to cross-validate the 16 possible relative configurations. Here, only NOE correlations between nuclei separated by more than five bonds and with a distance <5 Å were included in the analysis. We also selected two ^3^*J*_HH_ couplings, excluding all vicinal ^3^*J*_HH_ couplings to methyl or aromatic groups as they did not provide any stereochemical information (Table S5). These two couplings were used in combination with NOEs and ^13^C shifts for stereochemical assessment (Figure 3c). In this study, we exclusively used the ^13^C chemical shifts in the stereochemical assignment as the correlation between computed and experimental ^1^H chemical shifts was only intermediate. The results, as summarized in Figure 3, indicate that both NOEs and ^13^C chemical shifts support RC6 as the correct configuration, although NOEs provide less discrimination between the first- and second-ranking configurations. While the combination of the three types of isotropic NMR data supports RC6 as the correct configuration, the discrimination power of the isotropic NMR data is not as robust as the RDC analysis (Table S6). Notably, although the RDCs, NOEs, and ^13^C chemical shifts all point toward RC6 as the best-scoring configuration, the second-best ranking configuration suggested by all three analyses is significantly different.

We also attempted to use the acquired ΔΔRCSA data to assess the 16 possible relative configurations. Our previous studies have demonstrated the power of ΔΔRCSAs in stereochemical assignment, particularly for proton-deficient molecules.^17,23^ To our surprise, despite compound **1** also containing a quaternary carbon stereocenter (C3), ΔΔRCSAs provided very poor discrimination among the 16 relative configurations (Figure S12).

### Determination of the Absolute Configuration

**Figure 4:**
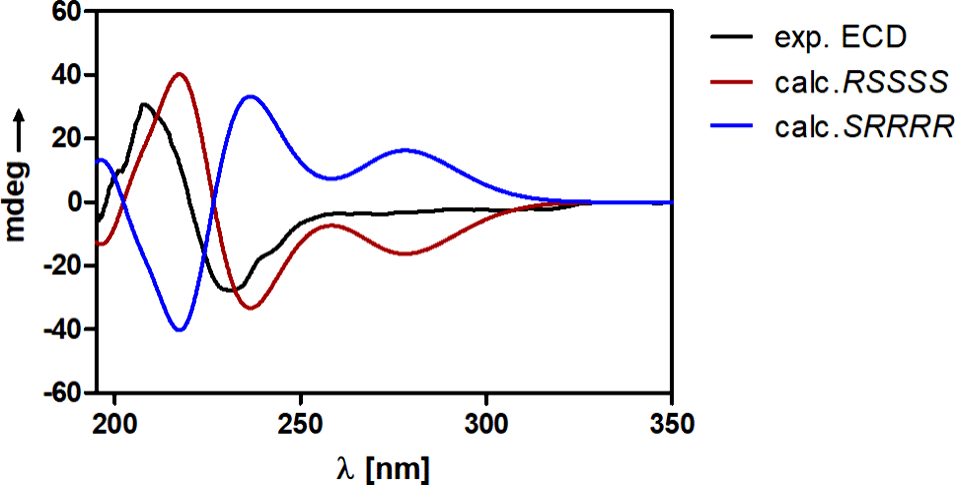
Comparison of the experimental (black) and computed *RSSSS* (red) and *SRRRR* (blue) ECD spectra of compound **1** in MeOH-*d*_4_ for determination of the absolute configuration.

**Figure 5:**
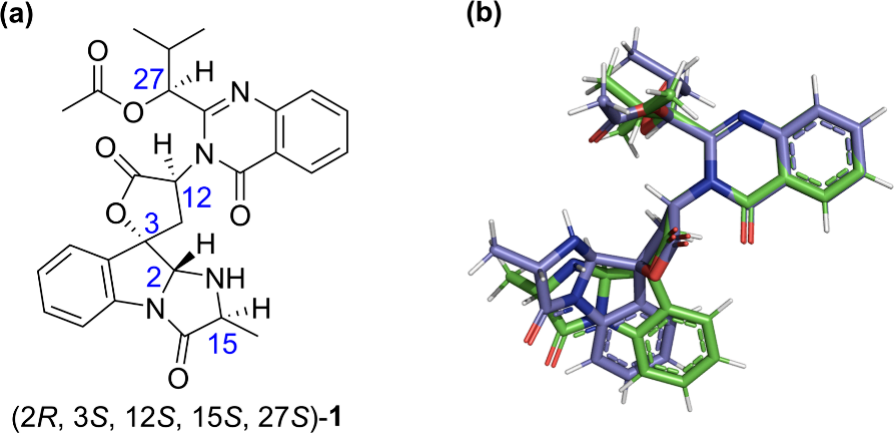
(a) The correct absolute configuration of compound **1** and (b) the determined structural ensemble of **1** from RDC analysis.

Finally, we determined the absolute configuration of compound **1** through a comparison between the experimental and back-calculated ECD spectra in MeOH-*d*_4_ (Figure 4). Experimental and computational details are provided in the Computational Methods. The experimental ECD spectrum displayed a key positive Cotton effect at 208 nm and a key negative Cotton effect at 230 nm, respectively. These spectral bands are well reproduced in the computed ECD spectra, thus resulting in an unambiguous determination of the absolute configuration of **1** as C2:*R*, C3:*S*, C12:*S*, C15:*S*, and C27:*S* (Figure 5). As compared to the originally identified deoxynortryptoquivaline,^3^ compound **1** possesses a different absolute configuration at C12. Thus, compound **1** was named 12*S*-deoxynortryptoquivaline as a new quinazolinone alkaloid.

## Conclusions

12*S*-Deoxynortryptoquivaline (**1**) falls within the category of challenging natural products for which determining the relative configuration using conventional NMR parameters proves to be difficult. We made attempts to leverage various types of isotropic and anisotropic NMR data to establish the relative configuration. Remarkably, the RDC analysis yielded the most discerning results among the 16 possible relative configurations. Although isotropic NMR data, including chemical shifts and NOEs, corroborated the results from the anisotropic NMR data, they did not yield the same level of distinctiveness as the RDC analysis. Finally, we conclusively determined the absolute configuration of 12*S*-deoxynortryptoquivaline (**1**) by comparison of the experimental and computed ECD spectra as C2:*R*, C3:*S*, C12:*S*, C15:*S*, and C27:*S*, with an ensemble comprising two primary conformers in the MeOH (Figure 5).

## Experimental Section

### General Experimental Procedures

NMR spectra were acquired on a Bruker AV750 MHz spectrometer using a 5 mm TCI Cryoprobe. Chemical shifts (δ) were referenced to the MeOH-*d*_4_ solvent to provide specific shifts. Oligopeptide AAKLVFF (purity >98%) was purchased from CSBio (Shanghai) Ltd. The 3 mm NMR tubes and MeOH-*d*_4_ (purity 99.8%) were purchased from Deutero GmbH. Mass spectra were recorded on an API QSTAR Pulsar 1 mass spectrometer (Applied Biosystems, Foster City, CA). UV spectra were read from a PuXi TU-1810 UV–visible spectrophotometer (Shanghai Lengguang Technology Co., Ltd., Shanghai, China). Column chromatography (CC) was used with silica gel (200–300 mesh, Qingdao Haiyang Chemical Factory, Qingdao, China), Lobar LiChroprep RP-18 (40–60 μm, Merck, Darmstadt, Germany), and Sephadex LH-20 (18–110 μm, Merck, Germany). Thin layer chromatography (TLC) was performed with silica gel GF254 precoated plates (100 × 200 mm, Qingdao Haiyang Chemical Group Corp., Qingdao, China). A separation and purification experiment was carried out with distilled organic solvents.

### Biological Material

The fungal strain *Aspergillus clavatus* AS-107 was isolated from the fresh tissue of a marine species of ascidian, which was collected from Lombok Island, Indonesia in October 2016. The fungus was identified as *A. clavatus* based on its ITS region sequence, which was found to be identical (100%) to that of *A. clavatus* TA30 (HQ392482). The sequence information on the fungus was deposited in GenBank with the accession no. MK785134. The strain is preserved at the Key Laboratory of Experimental Marine Biology, Institute of Oceanology, Chinese Academy of Sciences (IOCAS).

### Fermentation, Extraction, and Isolation

Fresh mycelia of *A. clavatus* AS-107 were grown on a PDA medium with seawater at 28 °C for 5 days and then inoculated into 98 × 1 L Erlenmeyer flasks with rice solid medium (70 g of rice, 0.3 g of peptone, 0.1 g of corn flour, and 100 mL of naturally sourced and filtered seawater), and statically cultured for 30 days at room temperature. The fermentation products were extracted three times with EtOAc, resulting in a crude extract of 115.7 g obtained through vacuum distillation. The crude extract was then fractionated by Si gel vacuum liquid chromatography (VLC), eluting with different solvents of increasing polarity from petroleum ether (PE) to MeOH to yield nine fractions (fractions 1–9). Fraction 5 (PE–EtOAc 1:1, 12.3 g) was further segmented by column chromatography (CC) over Lobar LiChroprep RP-18 with a MeOH–H_2_O gradient (from 10:90 to 100:0) to yield fractions 5.1–5.12. Fraction 5.8 (57.0 mg) was further purified by CC on Si gel eluting with a CH_2_Cl_2_–MeOH gradient (from 200:1 to 20:1) and then by Sephadex LH-20 eluting with MeOH to obtain compound **1** (5.2 mg).

#### 12*S*-Deoxynortryptoquivaline (**1**)

White amorphous powder; [α]^25^_D_ −168 (*c* 0.38, MeOH); UV (MeOH) λ_max_ (log ε) 228 (4.54), 265 (4.03), 317 (3.53) nm; ECD (0.54 mM, MeOH-*d*_4_) λ_max_ (Δε) 208 (+18.04), 230 (−15.78) nm; ^1^H and ^13^C NMR data, Table 1; HRESIMS *m*/*z* 517.2079 [M + H]^+^ (calcd for C_28_H_29_O_6_N_4_, 517.2082).

### Measurement of the Isotropic NMR Spectra

Compound **1** (1.5 mg) was dissolved in 250 μL of MeOH-*d*_4_ in a 3 mm NMR tube. 1D ^1^H, ^13^C and 2D HSQC, HMBC, COSY, NOESY, and CLIP-HSQC experiments were acquired at 300 K at a 750 MHz Bruker NMR spectrometer (750 and 187.5 MHz for ^1^H and ^13^C, respectively) that was equipped with a 5 mm TCL cryoprobe (z-gradient). All experiments used original Bruker pulse sequences, aside from the CLIP-HSQC, which used a home-modified pulse program. The number of scans for the above experiments was 24, 4000, 22, 30, 16, 24, and 26, respectively. The mixing time of the NOESY was 74 μs.

### Measurement of RDC and ΔΔRCSA Data of Compound **1**

After the acquisition of the above parameters, the AAKLVFF oligopeptide (7.2 mg) was then added to the solution, and the tube was inverted 1 time to achieve proper mixing and obtain the initial alignment condition. The sample was then stored at 3.8 °C and inverted 8 times per day. After 4 days, the aligned phase reached the equilibrated state (Δν_*Q*_(^2^H) = 13.5 Hz). The acquisition of 1D ^13^C and 2D CLIP-HSQC experiments was carried out under both initial and equilibrated alignment conditions. A total of 18 RDCs and 26 ΔΔRCSAs were obtained. To ensure the accuracy of their measurement, the RDC data were extracted five times from the same spectra, and the averaged value and standard deviation were derived from these five measurements.

### Measurement of ECD Spectrum

A CD spectrum was measured on a JASCO J-720 instrument. Compound **1** was dissolved in MeOH-*d*_4_ to a concentration of 5.38 × 10^–4^ mol/L. The applied acquisition parameters are as follows: cuvette of 0.1 cm path-length, 200 μL sample solutions, baseline correction, room temperature, data pitch (0.1 nm), scanning mode (continuous), wavelength range (180–400 nm), and accumulation (5).

### Computational Methods

A conformer ensemble was generated for each of the 16 possible relative configurations using the GMMX add-on module in GaussView 6. DFT optimization and energy calculation of the conformers were performed at the B3LYP/6-31G+* level with solvation modeled using IEFPCM and MeOH parameters in the program *Gaussian 16*.^24^ All conformers with Boltzmann population ≤5% in the energy calculations were discarded, and NMR calculations were performed on the remainder at the GIAO/B3LYP/6-311+G* level using IEFPCM MeOH solvation. The selected conformers of each relative configuration were aligned in *PyMOL*.^25^ Fitting of the NOEs, ^13^C chemical shifts, ^3^*J*_HH_ couplings, and ^1^*D*_CH_ data to each conformational ensemble of the 16 possible configurations was performed in *StereoFitter*, a Plug-in for MNova. Due to some technical issues in the current version of *StereoFitter*, MSpin was used for the ΔΔRCSA analysis and calculation of the *Q* factor. ECD spectra were simulated for the RDC-selected conformers (rc6_4 and rc6_6) under time-dependent density functional theory at the B3LYP/6-311+G* level using MeOH solvent. The calculations were carried out in *Gaussian16*.

## Supporting Information Available

General information on experimental procedures, characterization data, and NMR spectra for compound **1** (PDF)
Additional NMR details (ZIP)
