# Supplementary material for: Absolute Configuration of 12S-Deoxynortryptoquivaline from Ascidian-Derived Fungus Aspergillus clavatus Determined by Anisotropic NMR and Chiroptical Spectroscopy
Source: J Nat Prod. 2024 Jan 30;87(2):381–7. doi: 10.1021/acs.jnatprod.3c01157 (PMC10897928; doi:10.1021/acs.jnatprod.3c01157)
Supplement: Supplementary file 1 — np3c01157_si_001.pdf [file np3c01157_si_001.pdf]

Absolute Configuration of 12*S*-deoxynortryptoquivaline from Ascidian-derived Fungus *Aspergillus clavatus* Determined by Anisotropic NMR and Chiroptical Spectroscopy

Elisa Doro-Goldsmith[1,2#], Qi Song[3#], Xiaolu Li[1], Xiao-Ming Li[3], Xue-Yi Hu[3], Hong-Lei Li[3], Haoran Liu[1,4], Bin-Gui Wang[3,5*], Han Sun[1,4*]

[1]Leibniz-Forschungsinstitut für Molekulare Pharmakologie (FMP), Robert-Rössle-Str. 10, 13125, Germany

[2]School of Chemistry, The University of Edinburgh, David Brewster Road, Edinburgh, EH9 3FJ, United Kingdom

[3]CAS and Shandong Province Key Laboratory of Experimental Marine Biology, Institute of Oceanology, Chinese Academy of Sciences, Nanhai Road 7, Qingdao 266071, China

[4]Technische Universität Berlin, Institute of Chemistry, Straße des 17. Juni 135, 10623, Berlin, Germany

[5]University of Chinese Academy of Sciences, Yuquan Road 19A, Beijing 100049, China

# These authors contributed equally.

Correspondence: wangbg@ms.qdio.ac.cn (B.-G.W.), hsun@fmp-berlin.de (H.S.)

Supplemental Information

1. HRESI MASS SPECTRUM AND NMR SPECTRA OF COMPOUND **1** 3

2. SUPPLEMENTARY NOE, *J*-COUPLING, RDC AND ΔΔRCSA DATA AND ANALYSIS OF COMPOUND **1** 12

3. 3D-COORDINATES OF MAIN CONFORMERS FOR THE CORRECT CONFIGURATION OF COMPOUND **1** 21

4. OUTPUT OF THE STEREOFITTER CALCULATIONS FOR THE CORRECT CONFIGURATION OF COMPOUND **1** 27

1. HRESI mass spectrum and NMR spectra of compound 1

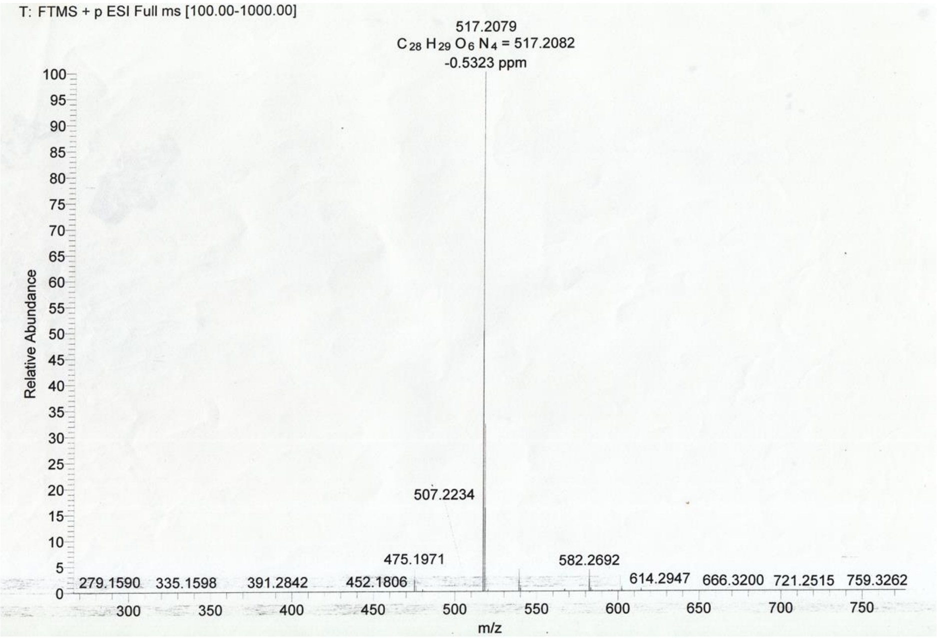

Figure S1. HRESI mass spectrum of compound **1**.

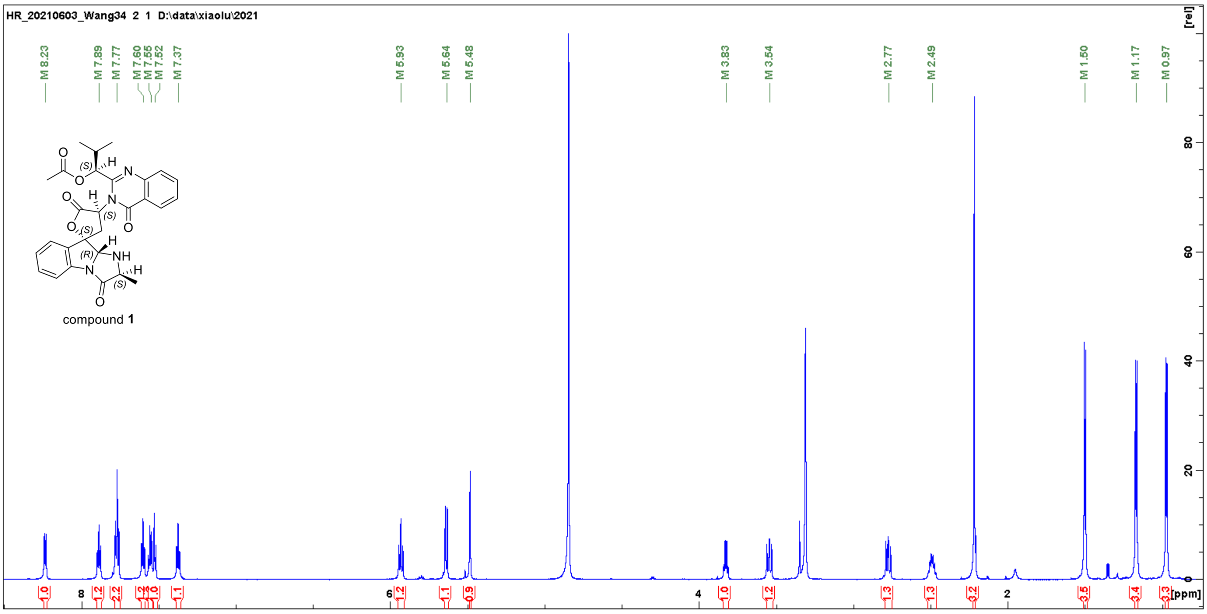

Figure S2. The ^{1}H NMR spectrum of compound **1** at 300 K (750 MHz, MeOH-d_4).

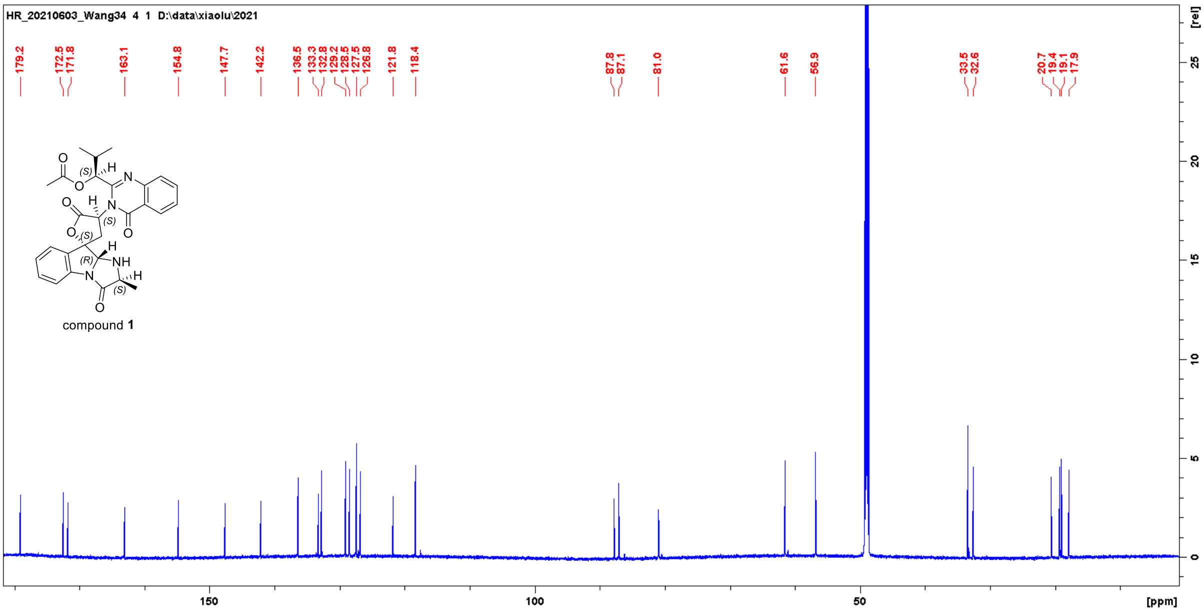

Figure S3. The ^{13}C NMR spectrum of compound **1** at 300 K (187.5 MHz, MeOH-d_4).

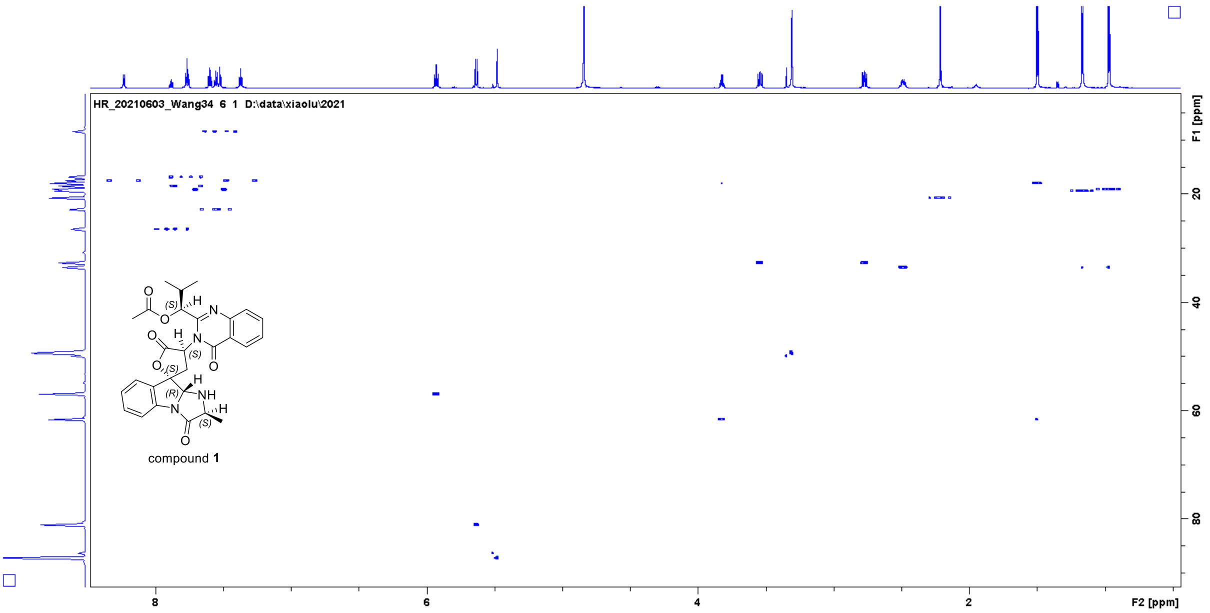

Figure S4. The ^{1}H-^{13}C HSQC spectrum of compound **1** at 300 K (750 and 187.5 MHz for ^{1}H and ^{13}C nuclei in MeOH-d_4, respectively).

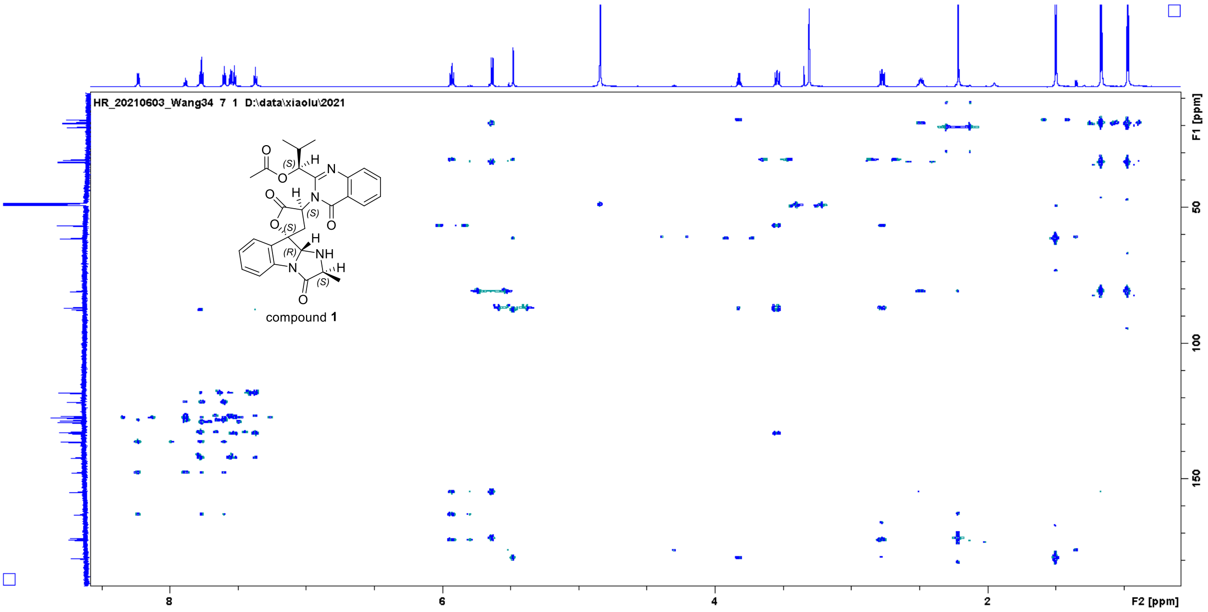

Figure S5. The ^{1}H-^{13}C HMBC spectrum of compound **1** at 300 K (750 and 187.5 MHz for ^{1}H and ^{13}C nuclei in MeOH-d_4, respectively).

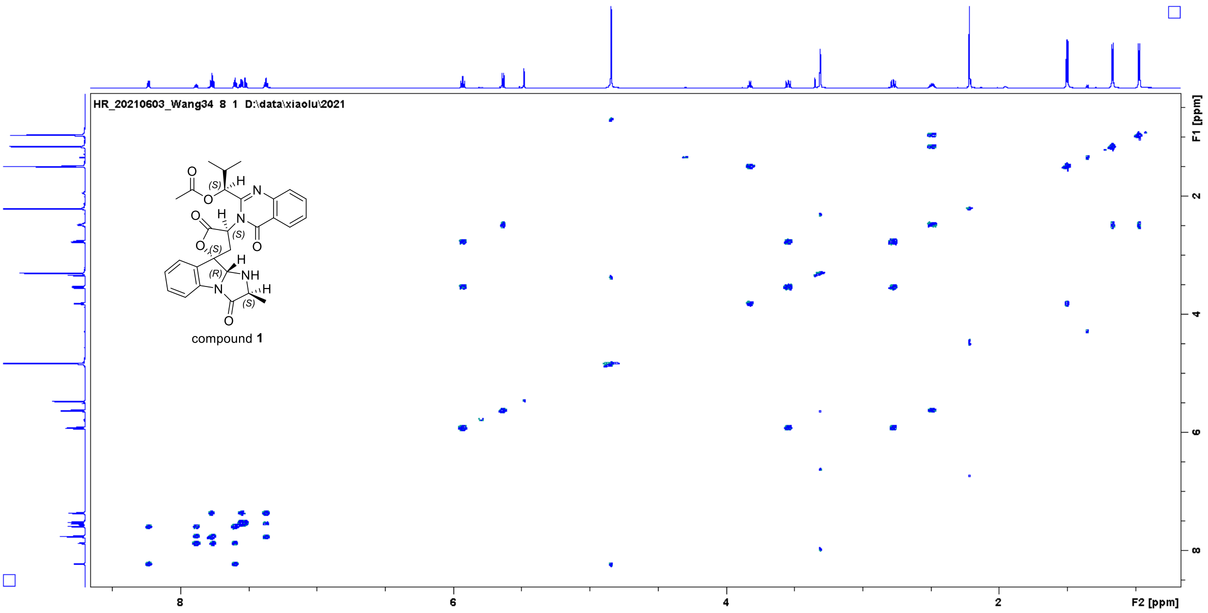

Figure S6. The ^{1}H-^{1}H COSY spectrum of compound **1** at 300 K (750 MHz, MeOH-d_4).

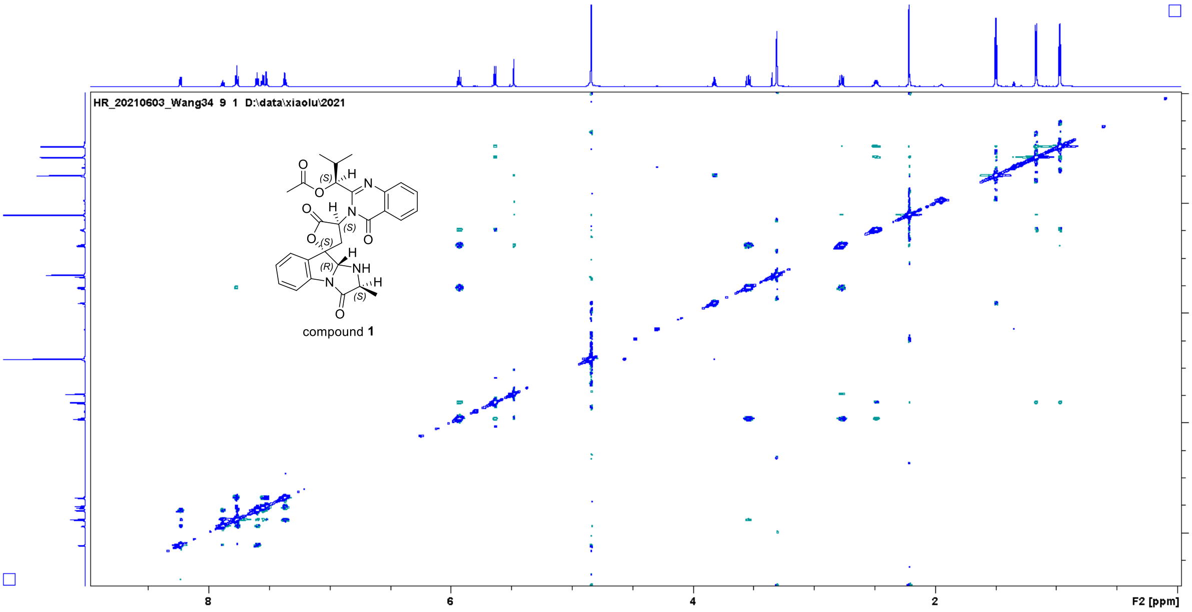

Figure S7. The ^1H-^1H NOESY spectrum of compound **1** at 300 K (750 MHz, MeOH-d_4,).

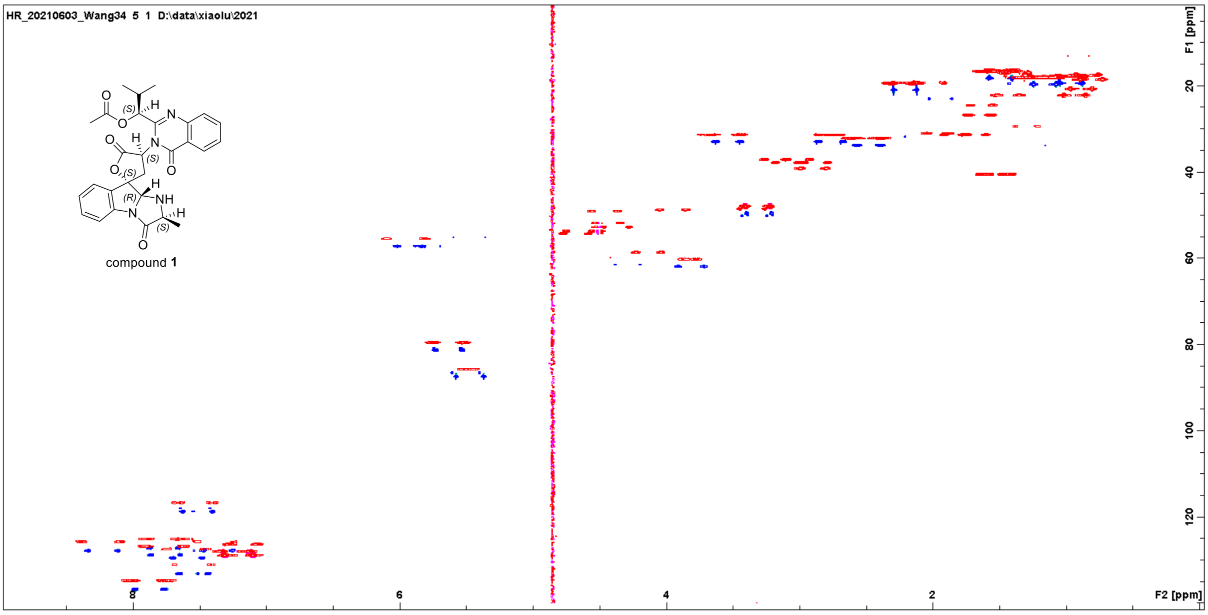

Figure S8. 2D ^{1}H-^{13}C CLIP-HSQC spectra of compound **1** under isotropic condition (blue) and equilibrated anisotropic condition (red) at 300 K (750 and 187.5 MHz for ^{1}H and ^{13}C nuclei in MeOH-d_4, respectively).

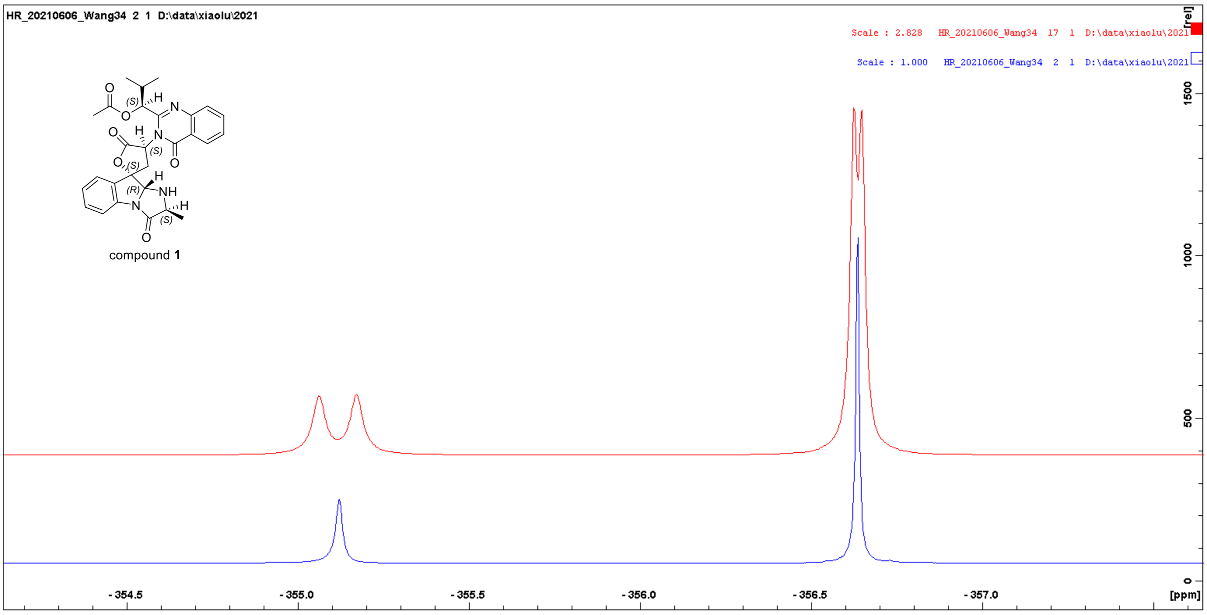

Figure S9. The ^{2}H spectra of compound **1** under initial (blue) and equilibrated (red) anisotropic conditions at 300 K (750 MHz, MeOH-d_4,).

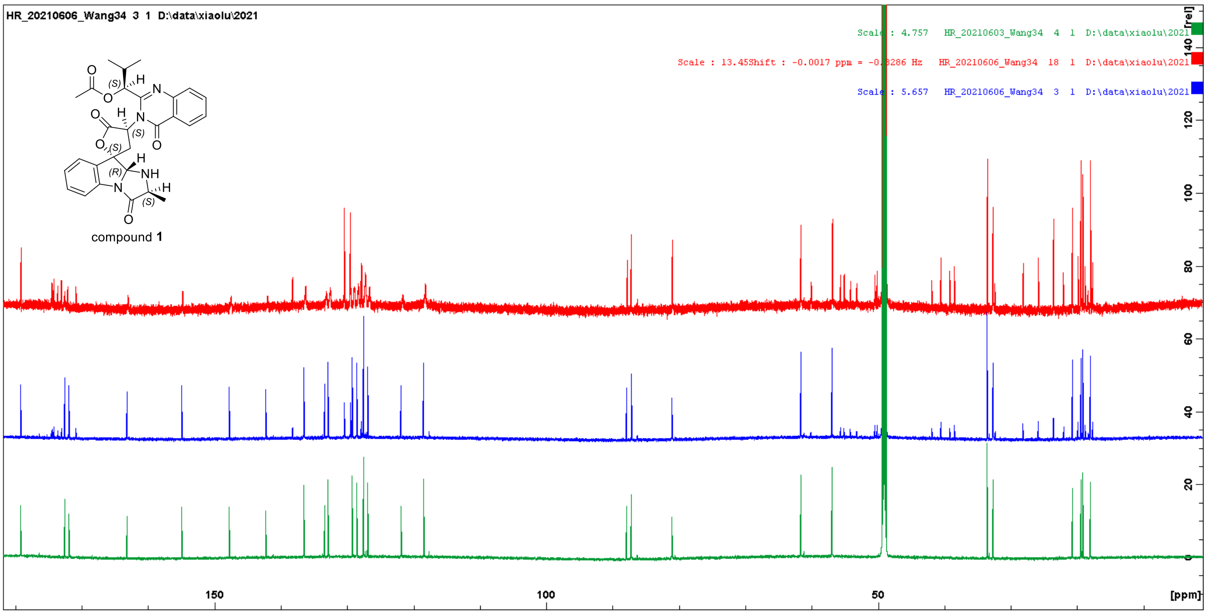

Figure S10. 1D ^{13}C spectra of compound **1** under isotropic condition (green), initial (blue) and equilibrated anisotropic condition (red) at 300 K (750 MHz, MeOH-d_4). All^{13}C spectra aligned by using carbon of MeOH-d_4 as reference atom.

2. Supplementary NOE, *J*-coupling, RDC and ΔΔRCSA data and analysis of compound 1

Table S1. The NOE correlations for compound **1** and the derived distances. A reference distance of 1.78 Å was used between geminal protons H13a and H13b. The row in grey indicates an NOE that was discarded from subsequent analysis due to a high uncertainty value.

Spin 1	Spin 2	Avg. intensity	Distance/Å
34	2	0.0065	4.1
33	13	0.0119	3.7
33	27	0.0108	3.8
28	12	0.0098	3.9
13 (δ= 3.54 ppm)	5	0.0171	3.5
2	33	0.0018	5.1
27	12	0.0388	3.1
12	33	0.0024	4.9
12	2	0.0832	2.7
5	13 (δ= 3.54 ppm)	0.0171	3.5

Table S2. RDC values for compound **1** in MeOD-d_4 using AAKLVFF (28.8 mg/mL) as the alignment medium. The average and standard deviation were calculated based on five independent measurements. The row in grey indicates an RDC that could not be measured due to poor resolution of the signal.

**Atom**	**δ(^{1}H)/ppm**	**δ(^{13}C)/ppm**	**Avg. $^1D_{CH}$/Hz**	**Std. dev. $^1D_{CH}$/Hz**
29 or 30	0.97	19.1	1.0	0.1
29 or 30	1.17	19.4	-14.6	1.1
34	1.50	17.9	-18.9	0.7
33	2.22	20.7	-13.1	1.0
28	2.49	33.5	31.7	0.9
13	2.78	32.6		
13	3.54	32.6	32.9	2.9
15	3.83	61.6	-71.3	2.4
2	5.48	87.1	-91.2	1.0
27	5.64	81.0	20.9	1.2
12	5.93	56.9	73.8	1.8
6	7.37	127.5	-2.7	0.7
8	7.53	118.4	29.4	1.7
7	7.56	132.8	66.8	2.6
21	7.60	129.2	57.1	2.5
23	7.76	128.5	54.8	1.8
5	7.78	126.8	26.3	2.6
22	7.89	136.5	36.3	1.9
20	8.24	127.5	46.8	0.9

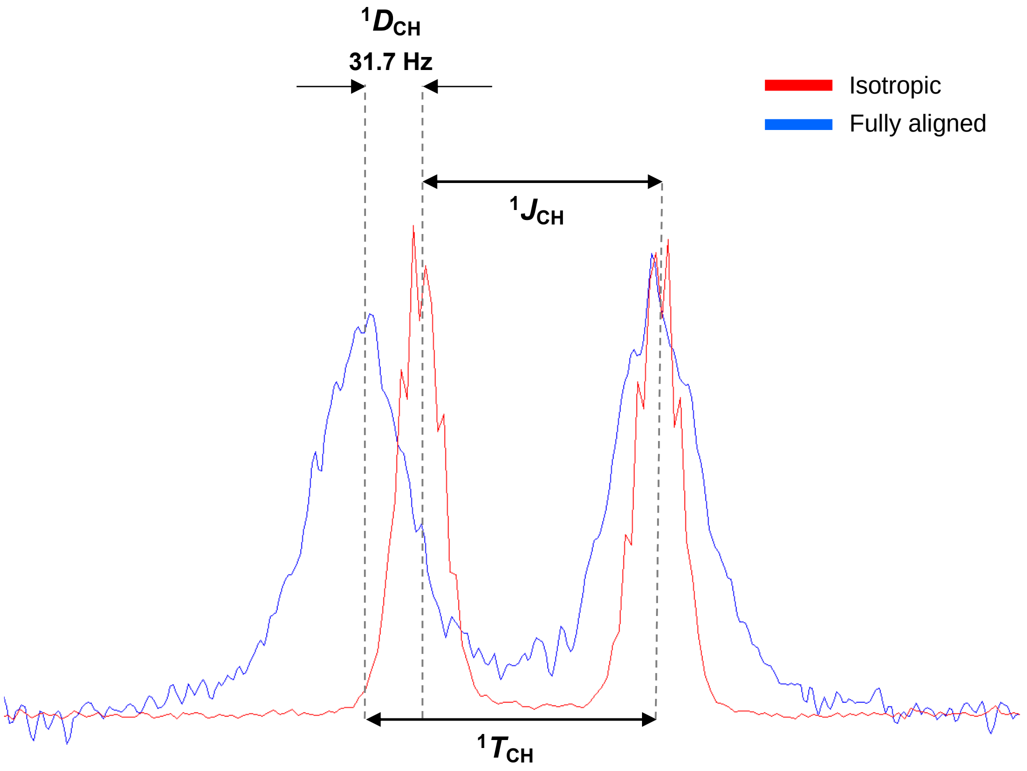

Figure S11. An example showing extraction of the ${}^{1}D_{CH}$ between atoms C28 and H28 at $\delta({}^{1}H)$ = 2.49 ppm (750 and 187.5 MHz for ${}^{1}H$ and ${}^{13}C$ nuclei in MeOH-d_4, respectively).

Table S3. The ΔΔRCSA values for compound **1** in MeOD-d_4 using AAKLVFF (28.8 mg/mL) as the alignment medium. The ΔΔRCSA values were extracted from two anisotropic ^{13}C spectra measured on days 1 and 4, using (i) C-28; (ii) C-12; and (iii) MeOD-d_4 as the reference signal on each occasion.

		**C-28**		**C-12**		**MeOD-*d*4**	
**Atom**	**δ(13 C)/ ppm**	**Avg./ ppm**	**Std. Dev./ ppm**	**Avg. / ppm**	**Std. Dev./ ppm**	**Avg./ ppm**	**Std. Dev./ ppm**
34	17.9	0.0045	0.0003	0.0488	0.0003	-0.0132	0.0004
29 or 30	19.1	0.0180	0.0003	0.0628	0.0003		
29 or 30	19.4	0.0259	0.0006	0.0709	0.0004	0.0079	0.0003
33	20.7	0.0204	0.0003	0.0651	0.0004	0.0032	0.0004
13	32.6	0.0164	0.0005	0.0611	0.0004	0.0015	0.0004
28	33.5			0.0444	0.0004	-0.0177	0.0004
12	56.9	-0.0475	0.0003			-0.0649	0.0006
15	61.6	0.0298	0.0006	0.0745	0.0006	0.0122	0.0004
27	81.0	-0.0327	0.0004	0.0132	0.0005	-0.0493	0.0005
2	87.1	0.0319	0.0006	0.0785	0.0005	0.0159	0.0006
3	87.8	-0.0768	0.0006	-0.0325	0.0005	0.0940	0.0006
8	118.4	-0.2573	0.0011	-0.2094	0.0028	-0.2714	0.0012
19	121.8	-0.2464	0.0031	-0.1972	0.0030	-0.2591	0.0041
5	126.8	-0.2521	0.0041	-0.2020	0.0029	-0.2620	0.0037
6 and 20	127.5	-0.2995	0.0010	-0.2498	0.0027	-0.3151	0.0021
23	128.5	-0.2311	0.0030	-0.1849	0.0011	-0.2490	0.0020
21	129.2	-0.3001	0.0035	-0.2515	0.0019	-0.3138	0.0019
7	132.8	-0.3322	0.0037	-0.2933	0.0036	-0.3491	0.0025
4	133.3	-0.2410	0.0042	-0.2005	0.0049	-0.2588	0.0041
22	136.5	-0.2549	0.0025	-0.2099	0.0031	-0.2721	0.0038

9	142.2	-0.2698	0.0030	-0.2245	0.0023	-0.2901	0.0033
24	147.7	-0.2654	0.0026	-0.2198	0.0018	-0.2825	0.0027
26	154.8	-0.1427	0.0014	-0.0975	0.0007	-0.1611	0.0009
18	163.1	-0.1765	0.0012	-0.1316	0.0016	-0.1944	0.0013
32	171.8	0.2076	0.0024	0.2557	0.0018	0.1924	0.0015
11	172.5	-0.0223	0.0024	0.0305	0.0015	-0.0387	0.0017
14	179.2	-0.0402	0.0005	0.0053	0.0005	-0.0571	0.0006

Table S4. Number of conformers for each possible relative configuration of compound **1** from a molecular mechanics-based conformational search and density functional theory-based structural optimization. The relative configuration is numbered C2, C3, C12, C15, C27.

**Name**	**Relative Configuration**	**No. Conformers**
RC1	*RRSRS*	12
RC2	*RSSRS*	6
RC3	*RRRRS*	8
RC4	*RSRRS*	7
RC5	*RRSSS*	11
RC6	*RSSSS*	10
RC7	*RRRSS*	8
RC8	*RSRSS*	7
RC9	*SRSRS*	5
RC10	*SSSRS*	3
RC11	*SRRRS*	8
RC12	*SSRRS*	19
RC13	*SRSSS*	5
RC14	*SSSSS*	3
RC15	*SRRSS*	7
RC16	*SSRSS*	14

Table S5. ${}^3J_{HH}$ couplings of compound **1** used in cross-validation against 16 possible relative configurations.

**Atom**	**δ(^{1}H)/ppm**	**Avg./Hz**	**Std. dev./Hz**
27	5.64	9.7	0.1
28	2.49	6.7	0.1

Table S6. AIC values for the three lowest-scoring relative configurations of compound **1** for different combinations of $^{3}J_{HH}$ couplings, NOEs, and ^{13}C chemical shifts.

**Parameter Combination**	**1st Lowest-scoring/AIC**	**2nd Lowest-scoring/AIC**	**3rd Lowest-scoring/AIC**
NOEs	RC6, 38.9	RC16, 40.4	RC4, 40.6
^{13}C shifts	RC6, 25.4	RC9, 34.1	RC8, 34.9
$^{3}J_{HH}$ + NOEs + ^{13}C shifts	RC6, 84.0	RC8, 90.2	RC4, 93.2

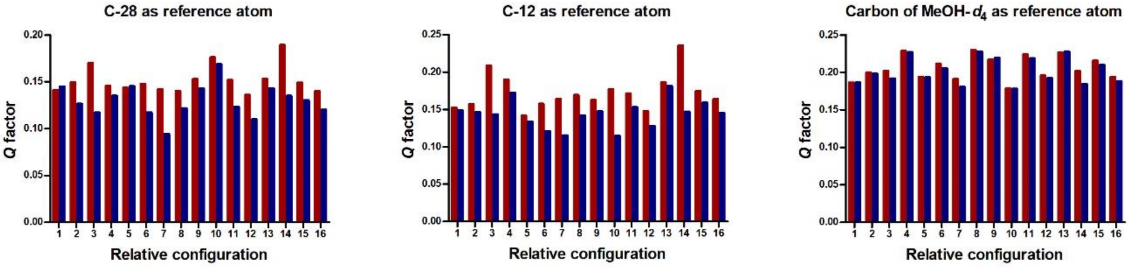

Figure S12. The ΔΔRCSA analysis for compound **1** with reference atoms C-28, C-12 and carbon of MeOH-d_4, respectively. Red bars represent results when all 26 ΔΔRCSAs were employed, while blue bars show the results excluding ΔΔRCSA values of C-32 and C-33 located in a flexible acetyl group.

3. 3D-coordinates of main conformers for the correct configuration of compound 1

Coordinates for conformer 4 of relative configuration 6 (C2:*R, C3:*S**, C12:*S**, C15:*S**, C27:*S**) of compound 1**

HETATM	1	C	0	4.301	1.241	0.118	C
HETATM	2	C	0	3.269	2.170	-0.137	C
HETATM	3	C	0	3.551	3.547	-0.214	C
HETATM	4	C	0	4.852	3.993	-0.035	C
HETATM	5	C	0	5.886	3.069	0.223	C
HETATM	6	C	0	5.619	1.709	0.299	C
HETATM	7	N	0	4.055	-0.118	0.194	N
HETATM	8	C	0	2.848	-0.561	0.026	C
HETATM	9	N	0	1.755	0.275	-0.206	N
HETATM	10	C	0	1.912	1.673	-0.312	C
HETATM	11	O	0	0.922	2.376	-0.536	O
HETATM	12	C	0	2.712	-2.083	0.057	C
HETATM	13	O	0	1.660	-2.481	0.979	O
HETATM	14	C	0	2.467	-2.770	-1.307	C
HETATM	15	H	0	3.663	-2.431	0.460	H
HETATM	16	C	0	3.513	-2.320	-2.340	C
HETATM	17	C	0	2.491	-4.297	-1.136	C
HETATM	18	H	0	6.406	0.988	0.494	H
HETATM	19	H	0	6.903	3.426	0.362	H
HETATM	20	H	0	2.741	4.242	-0.412	H
HETATM	21	H	0	5.076	5.054	-0.092	H
HETATM	22	C	0	2.011	-2.882	2.232	C
HETATM	23	C	0	0.800	-3.260	3.041	C
HETATM	24	O	0	3.160	-2.912	2.628	O
HETATM	25	C	0	0.374	-0.208	-0.337	C
HETATM	26	C	0	-0.295	0.285	-1.623	C
HETATM	27	O	0	-1.591	0.575	-1.374	O
HETATM	28	C	0	-1.940	0.441	0.054	C
HETATM	29	C	0	-0.595	0.211	0.780	C
HETATM	30	H	0	0.401	-1.292	-0.405	H

HETATM	31	O	0	0.179	0.335	-2.734		O
HETATM	32	C	0	-2.753	1.660	0.456		C
HETATM	33	C	0	-4.124	1.352	0.488		C
HETATM	34	N	0	-4.295	-0.012	0.208		N
HETATM	35	C	0	-2.988	-0.702	0.216		C
HETATM	36	C	0	-5.244	-0.736	-0.455		C
HETATM	37	C	0	-4.542	-2.043	-0.891		C
HETATM	38	N	0	-3.086	-1.738	-0.802		N
HETATM	39	C	0	-2.324	2.962	0.695		C
HETATM	40	C	0	-3.282	3.946	0.973		C
HETATM	41	C	0	-4.646	3.624	1.001		C
HETATM	42	C	0	-5.091	2.318	0.761		C
HETATM	43	O	0	-6.412	-0.412	-0.660		O
HETATM	44	C	0	-4.954	-3.233	-0.017		C
HETATM	45	H	0	3.343	-2.846	-3.286		H
HETATM	46	H	0	3.462	-1.245	-2.544		H
HETATM	47	H	0	4.530	-2.554	-2.002		H
HETATM	48	H	0	2.303	-4.782	-2.100		H
HETATM	49	H	0	3.471	-4.635	-0.775		H
HETATM	50	H	0	1.728	-4.642	-0.431		H
HETATM	51	H	0	1.106	-3.862	3.898		H
HETATM	52	H	0	-1.268	3.214	0.651		H
HETATM	53	H	0	-2.965	4.967	1.168		H
HETATM	54	H	0	-5.376	4.400	1.217		H
HETATM	55	H	0	-6.145	2.065	0.785		H
HETATM	56	H	0	1.476	-2.486	-1.682		H
HETATM	57	H	0	-2.846	-1.152	1.207		H
HETATM	58	H	0	-6.037	-3.384	-0.069		H
HETATM	59	H	0	-4.456	-4.141	-0.372		H
HETATM	60	H	0	-4.678	-3.076	1.033		H
HETATM	61	H	0	0.073	-3.804	2.433		H
HETATM	62	H	0	0.316	-2.347	3.409		H
HETATM	63	H	0	-0.685	-0.560	1.547		H
HETATM	64	H	0	-0.262	1.131	1.259		H

HETATM 65 H 0 -4.806 -2.248 -1.933 H
HETATM 66 H 0 -2.794 -1.332 -1.691 H
END

Coordinates for conformer 6 of relative configuration 6 (C2:*R, C3:*S**, C12:*S**, C15:*S**, C27:*S**) of compound 1**

HETATM	1	C	0	4.378	-1.144	0.203	C
HETATM	2	C	0	3.432	-2.145	0.514	C
HETATM	3	C	0	3.857	-3.428	0.908	C
HETATM	4	C	0	5.213	-3.706	0.999	C
HETATM	5	C	0	6.161	-2.708	0.695	C
HETATM	6	C	0	5.753	-1.442	0.299	C
HETATM	7	N	0	3.991	0.115	-0.218	N
HETATM	8	C	0	2.734	0.409	-0.281	C
HETATM	9	N	0	1.721	-0.485	0.078	N
HETATM	10	C	0	2.014	-1.819	0.432	C
HETATM	11	O	0	1.093	-2.608	0.660	O
HETATM	12	C	0	2.362	1.809	-0.777	C
HETATM	13	O	0	2.354	2.656	0.415	O
HETATM	14	C	0	3.348	2.378	-1.814	C
HETATM	15	H	0	1.363	1.826	-1.211	H
HETATM	16	C	0	3.302	1.534	-3.100	C
HETATM	17	C	0	3.029	3.849	-2.128	C
HETATM	18	H	0	6.473	-0.666	0.056	H
HETATM	19	H	0	7.221	-2.933	0.769	H
HETATM	20	H	0	3.112	-4.182	1.140	H
HETATM	21	H	0	5.546	-4.693	1.306	H
HETATM	22	C	0	1.246	3.377	0.712	C
HETATM	23	C	0	1.469	4.239	1.925	C
HETATM	24	O	0	0.211	3.313	0.070	O
HETATM	25	C	0	0.302	-0.114	0.109	C
HETATM	26	C	0	-0.406	-0.550	1.401	C
HETATM	27	O	0	-1.664	-0.931	1.105	O
HETATM	28	C	0	-1.965	-0.717	-0.320	C
HETATM	29	C	0	-0.590	-0.691	-0.999	C
HETATM	30	H	0	0.231	0.974	0.114	H
HETATM	31	O	0	0.020	-0.507	2.533	O
HETATM	32	C	0	-2.987	-1.752	-0.740	C

HETATM	33	C	0	-4.278	-1.201	-0.661		C
HETATM	34	N	0	-4.181	0.165	-0.316		N
HETATM	35	C	0	-2.794	0.610	-0.477		C
HETATM	36	C	0	-4.866	0.894	0.619		C
HETATM	37	C	0	-3.935	2.019	1.084		C
HETATM	38	N	0	-2.619	1.665	0.505		N
HETATM	39	C	0	-2.821	-3.091	-1.077		C
HETATM	40	C	0	-3.960	-3.868	-1.334		C
HETATM	41	C	0	-5.240	-3.305	-1.242		C
HETATM	42	C	0	-5.421	-1.957	-0.906		C
HETATM	43	O	0	-6.005	0.674	1.020		O
HETATM	44	C	0	-4.446	3.409	0.685		C
HETATM	45	H	0	4.010	1.937	-3.833		H
HETATM	46	H	0	3.570	0.488	-2.918		H
HETATM	47	H	0	2.303	1.560	-3.554		H
HETATM	48	H	0	3.738	4.225	-2.874		H
HETATM	49	H	0	2.019	3.961	-2.541		H
HETATM	50	H	0	3.113	4.487	-1.243		H
HETATM	51	H	0	1.755	3.615	2.778		H
HETATM	52	H	0	-1.831	-3.535	-1.133		H
HETATM	53	H	0	-3.849	-4.913	-1.605		H
HETATM	54	H	0	-6.112	-3.921	-1.443		H
HETATM	55	H	0	-6.410	-1.517	-0.845		H
HETATM	56	H	0	4.354	2.316	-1.388		H
HETATM	57	H	0	-2.640	0.960	-1.508		H
HETATM	58	H	0	-5.428	3.592	1.134		H
HETATM	59	H	0	-3.758	4.182	1.047		H
HETATM	60	H	0	-4.534	3.502	-0.404		H
HETATM	61	H	0	0.557	4.790	2.158		H
HETATM	62	H	0	2.289	4.940	1.739		H
HETATM	63	H	0	-0.594	-0.080	-1.905		H
HETATM	64	H	0	-0.268	-1.701	-1.259		H
HETATM	65	H	0	-3.894	1.959	2.180		H
HETATM	66	H	0	-2.142	2.471	0.111		H

END

4. Output of the StereoFitter calculations for the correct configuration of compound 1

Output of StereoFitter calculations for relative configuration 6 using NOE data

Solution 1 (2 conformers), χ^2: 36.884, AIC: 38.884, Relative Probability: 1.000

NOE distances (Å)

Atoms Experimental Calculated Average

57,(58,59,60) 4,120 3,592

15,(51,61,62) 3,790 4,438

30.56 3,850 2,718

63.64 1,810 1,773

15.3 3,060 2,003

30,(51,61,62) 4,860 4,268

30.57 2,690 3,342

Output of StereoFitter calculations for relative configuration 6 using ^{13}C chemical shift data

Solution 1 (1 conformers), χ^2: 25.412, AIC: 25.412, Relative Probability: 1.000

Chemical shifts (ppm)

Atoms	Experimental	Calculated Average
35	87,100	86,961
28	87,800	88,125
32	133,300	132,544
39	126,800	125,715
40	127,500	125,226
41	132,800	131,658
42	118,400	115,898
33	142,200	140,984
26	172,500	173,413
25	56,900	57,399
29	32,600	34,141
36	179,200	174,238
37	61,600	65,044
10	163,100	162,108
2	121,800	120,852
3	127,500	126,350
4	129,200	127,734
5	136,500	135,177
6	128,500	127,633
1	147,700	146,736
8	154,800	153,422
12	81,000	77,050
14	33,500	34,646
16.17	19,300	17,237
22	171,800	175,233
23	20,700	20,328
44	17,900	19,257

Output of StereoFitter calculations for relative configuration 6 using NOE, ^{13}C chemical shift and $^{3}J_{HH}$ coupling data

Solution 1 (2 conformers), χ^2: 82.037, AIC: 84.037, Relative Probability: 1.000

NOE distances (Å)

Atoms	Experimental	Calculated Average
57,(58,59,60)	4,120	3,555
15,(51,61,62)	3,790	4,438
30.56	3,850	2,638
63.64	1,810	1,773
15.3	3,060	2,018
30,(51,61,62)	4,860	4,258
30.57	2,690	3,352

J couplings (Hz)

Atoms	Experimental	Calculated Average
15.56	9,660	10,540
30,(63,64)	9.410,10.330	7.348,9.971

Chemical shifts (ppm)

Atoms	Experimental	Calculated Average
35	87,100	86,956
28	87,800	87,882
32	133,300	132,962
39	126,800	125,976
40	127,500	125,103
41	132,800	131,739
42	118,400	115,445
33	142,200	140,581
26	172,500	173,474
25	56,900	57,343
29	32,600	34,769
36	179,200	174,347
37	61,600	65,488
10	163,100	162,159
2	121,800	120,728
3	127,500	126,364

4	129,200	127,742
5	136,500	135,247
6	128,500	127,653
1	147,700	146,758
8	154,800	153,416
12	81,000	78,520
14	33,500	35,093
16.17	19,300	17,442
22	171,800	174,910
23	20,700	20,327
44	17,900	18,617

Output of StereoFitter calculations for relative configuration 6 using RDC data

Solution 1 (2 conformers), χ^2: 1496.994, AIC: 1498.994, Relative Probability: 1.000

Conformer 2: 60.22%

Conformer 3: 39.78%

RDC couplings (Hz)

Atoms Experimental Calculated Average

(44,58) (44,59) (44,60) -18.88 0.736

(23,51) (23,61) (23,62) -13.11 0.881

14,56 31.68 24.673

37,65 -71.27 -87.689

35,57 -91.15 -68.841

12,15 20.94 22.667

25,30 73.78 26.263

40,53 -2.66 14.898

42,55 29.4 51.296

41,54 66.79 59.935

4,21 57.09 15.914

6,18 54.77 55.632

39,52 26.32 51.36

5,19 36.29 43.225

3,20 46.76 56.642

Solution 2 (2 conformers), χ^2: 1928.651, AIC: 1930.651, Relative Probability: 0.000

Conformer 1: 80.09%

Conformer 2: 19.91%

RDC couplings (Hz)

Atoms Experimental Calculated Average

(44,58) (44,59) (44,60) -18.88 -6.921

(23,51) (23,61) (23,62) -13.11 -24.408

14,56 31.68 32.55

37,65 -71.27 -121.323

35,57 -91.15 -72.794

12,15 20.94 26.098

25,30 73.78 43.759

40,53 -2.66 7.423

42,55 29.4 32.441

41,54 66.79 94.69

4,21 57.09 33.787

6,18 54.77 47.547

39,52 26.32 35.514

5,19 36.29 19.041

3,20 46.76 47.625

Solution 3 (1 conformers), χ^2: 2032.930, AIC: 2032.930, Relative Probability: 0.000

Conformer 1: 100%

RDC couplings (Hz)

Atoms Experimental Calculated Average

(44,58) (44,59) (44,60) -18.88 -6.6

(23,51) (23,61) (23,62) -13.11 -24.762

14,56 31.68 35.885

37,65 -71.27 -118.066

35,57 -91.15 -70.484

12,15 20.94 27.827

25,30 73.78 46.356

40,53 -2.66 7.614

42,55 29.4 25.868

41,54 66.79 96.954

4,21 57.09 34.581

6,18 54.77 47.837

39,52 26.32 28.988

5,19 36.29 17.867

3,20 46.76 48.052

Solution 4 (2 conformers), χ^2: 2031.306, AIC: 2033.306, Relative Probability: 0.000

Conformer 1: 98.13%

Conformer 3: 1.87%

RDC couplings (Hz)

Atoms Experimental Calculated Average

(44,58) (44,59) (44,60) -18.88 -6.654

(23,51) (23,61) (23,62) -13.11 -24.583

14,56 31.68 35.496

37,65 -71.27 -118.925

35,57 -91.15 -70.836

12,15 20.94 27.433

25,30 73.78 45.679

40,53 -2.66 7.707

42,55 29.4 26.67

41,54 66.79 96.476

4,21 57.09 33.701

6,18 54.77 47.754

39,52 26.32 29.799

5,19 36.29 18.584

3,20 46.76 48.026

Solution 5 (1 conformers), χ^2: 2626.839, AIC: 2626.839, Relative Probability: 0.000

Conformer 2: 100%

RDC couplings (Hz)

Atoms Experimental Calculated Average

(44,58) (44,59) (44,60) -18.88 -1.547

(23,51) (23,61) (23,62) -13.11 -16.168

14,56 31.68 21.629

37,65 -71.27 -105.406

35,57 -91.15 -71.462

12,15 20.94 19.589

25,30 73.78 33.432

40,53 -2.66 9.487

42,55 29.4 48.694

41,54 66.79 77.44

4,21 57.09 25.104

6,18 54.77 57.135

39,52 26.32 50.086

5,19 36.29 25.243

3,20 46.76 57.012

Solution 6 (1 conformers), χ^2: 3720.845, AIC: 3720.845, Relative Probability: 0.000

Conformer 3: 100%

RDC couplings (Hz)

Atoms	Experimental	Calculated Average
(44,58) (44,59) (44,60)	-18.88	-8.885
(23,51) (23,61) (23,62)	-13.11	14.934
14,56	31.68	37.577
37,65	-71.27	-78.274
35,57	-91.15	-62.685
12,15	20.94	38.332
25,30	73.78	33.738
40,53	-2.66	19.109
42,55	29.4	30.247
41,54	66.79	57.705
4,21	57.09	27.24
6,18	54.77	50.304
39,52	26.32	30.266
5,19	36.29	41.925
3,20	46.76	51.189

Output of StereoFitter calculations for relative configuration 6 using NOE, ^{13}C chemical shift, $^3J_{HH}$ coupling and RDC data

Solution 1 (2 conformers), χ^2: 1768.396, AIC: 1770.396, Relative Probability: 1.000

Conformer 2: 59.75%

Conformer 3: 40.25%

NOE distances (Å)

Atoms	Experimental	Calculated Average
57,(58,59,60)	4.12	3.281
15,(51,61,62)	3.79	4.433
30,56	3.85	2.235
63,64	1.81	1.77
15,30	3.06	2.244
30,(51,61,62)	4.86	4.165
30,57	2.69	3.467

RDC couplings (Hz)

Atoms	Experimental	Calculated Average
(44,58) (44,59) (44,60)	-18.88	0.75
(23,51) (23,61) (23,62)	-13.11	1.148
14,56	31.68	24.749
37,65	-71.27	-87.409
35,57	-91.15	-68.819
12,15	20.94	22.738
25,30	73.78	26.271
40,53	-2.66	14.945
42,55	29.4	51.243
41,54	66.79	59.779
4,21	57.09	15.988
6,18	54.77	55.59
39,52	26.32	51.294
5,19	36.29	43.395
3,20	46.76	56.605

Chemical shifts (ppm)

Atoms	Experimental	Calculated Average
35	87.1	86.949

57	5.48	5.143	
28	87.8	87.454	
32	133.3	133.701	
39	126.8	126.437	
52	7.78	7.412	
40	127.5	124.886	
53	7.37	6.915	
41	132.8	131.881	
54	7.56	7.138	
42	118.4	114.644	
55	7.53	7.05	
33	142.2	139.869	
26	172.5	173.582	
25	56.9	57.244	
30	5.93	4.879	
29	32.6	35.88	
63,64		3.16	2.721
36	179.2	174.54	
37	61.6	66.275	
65	3.83	3.574	
10	163.1	162.247	
2	121.8	120.509	
3	127.5	126.389	
20	8.24	7.803	
4	129.2	127.757	
21	7.6	7.165	
5	136.5	135.37	
19	7.89	7.452	
6	128.5	127.691	
18	7.76	7.221	
1	147.7	146.796	
8	154.8	153.405	
12	81	81.118	
15	5.64	5.284	

14 33.5 35.883

56 2.49 2.265

16,17 19.3 17.803

45,46,47,48,49,50 1.07 0.777

22 171.8 174.339

23 20.7 20.325

51,61,62 2.22 1.871

44 17.9 17.486

58,59,60 1.5 1.255

Solution 2 (2 conformers), χ^2: 2159.325, AIC: 2161.325, Relative Probability: 0.000

Conformer 1: 78.95%

Conformer 2: 21.05%

NOE distances (Å)

Atoms Experimental Calculated Average

57,(58,59,60) 4.12 3.101

15,(51,61,62) 3.79 4.43

30,56 3.85 2.649

63,64 1.81 1.766

15,30 3.06 1.922

30,(51,61,62) 4.86 4.65

30,57 2.69 3.672

RDC couplings (Hz)

Atoms Experimental Calculated Average

(44,58) (44,59) (44,60) -18.88 -6.917

(23,51) (23,61) (23,62) -13.11 -24.365

14,56 31.68 32.329

37,65 -71.27 -121.431

35,57 -91.15 -72.902

12,15 20.94 25.968

25,30 73.78 43.585

40,53 -2.66 7.422

42,55 29.4 32.851

41,54 66.79 94.501

4,21 57.09 33.711

6,18	54.77	47.577	
39,52		26.32	35.914
5,19	36.29	19.151	
3,20	46.76	47.649	

Chemical shifts (ppm)

Atoms		Experimental	Calculated Average
35	87.1	87.046	
57	5.48	5.097	
28	87.8	86.951	
32	133.3	134.746	
39	126.8	126.95	
52	7.78	7.581	
40	127.5	124.706	
53	7.37	6.914	
41	132.8	131.934	
54	7.56	7.11	
42	118.4	113.817	
55	7.53	7.037	
33	142.2	139.092	
26	172.5	173.708	
25	56.9	56.634	
30	5.93	5.558	
29	32.6	37.767	
63,64		3.16	2.791
36	179.2	174.919	
37	61.6	67.071	
65	3.83	3.519	
10	163.1	161.841	
2	121.8	120.48	
3	127.5	126.503	
20	8.24	7.819	
4	129.2	127.543	
21	7.6	7.168	
5	136.5	135.407	

19 7.89 7.446

6 128.5 127.445

18 7.76 7.217

1 147.7 147.065

8 154.8 155.279

12 81 78.856

15 5.64 4.969

14 33.5 37.034

56 2.49 2.356

16,17 19.3 17.665

45,46,47,48,49,50 1.07 0.763

22 171.8 174.923

23 20.7 20.08

51,61,62 2.22 1.803

44 17.9 16.312

58,59,60 1.5 1.245

Solution 3 (2 conformers), χ^2: 2288.961, AIC: 2290.961, Relative Probability: 0.000

Conformer 1: 97.27%

Conformer 3: 2.73%

NOE distances (Å)

Atoms Experimental Calculated Average

57,(58,59,60) 4.12 3.111

15,(51,61,62) 3.79 4.431

30,56 3.85 4.617

63,64 1.81 1.766

15,30 3.06 1.852

30,(51,61,62) 4.86 4.927

30,57 2.69 3.669

RDC couplings (Hz)

Atoms Experimental Calculated Average

(44,58) (44,59) (44,60) -18.88 -6.67

(23,51) (23,61) (23,62) -13.11 -24.495

14,56 31.68 35.304

37,65 -71.27 -119.313

35,57	-91.15	-71.002
12,15	20.94	27.24
25,30	73.78	45.355
40,53	-2.66	7.752
42,55	29.4	27.064
41,54	66.79	96.236
4,21	57.09	33.273
6,18	54.77	47.718
39,52	26.32	30.196
5,19	36.29	18.93
3,20	46.76	48.017

Chemical shifts (ppm)

Atoms	Experimental	Calculated Average
35	87.1	87.071
57	5.48	5.105
28	87.8	86.969
32	133.3	134.754
39	126.8	126.923
52	7.78	7.572
40	127.5	124.733
53	7.37	6.919
41	132.8	131.901
54	7.56	7.107
42	118.4	113.878
55	7.53	7.041
33	142.2	139.137
26	172.5	173.703
25	56.9	56.524
30	5.93	5.674
29	32.6	37.854
63,64	3.16	2.801
36	179.2	174.947
37	61.6	67.008
65	3.83	3.529

10	163.1	161.719
2	121.8	120.542
3	127.5	126.521
20	8.24	7.813
4	129.2	127.489
21	7.6	7.169
5	136.5	135.376
19	7.89	7.444
6	128.5	127.376
18	7.76	7.22
1	147.7	147.115
8	154.8	155.718
12	81	77.51
15	5.64	4.882
14	33.5	37.052
56	2.49	2.454
16,17	19.3	17.518
45,46,47,48,49,50	1.07	0.752
22	171.8	175.238
23	20.7	20.023
51,61,62	2.22	1.775
44	17.9	16.397
58,59,60	1.5	1.248

Solution 4 (1 conformers), χ^2: 2292.546, AIC: 2292.546, Relative Probability: 0.000

Conformer 1: 100%

NOE distances (Å)

Atoms	Experimental	Calculated Average
57,(58,59,60)	4.12	3.101
15,(51,61,62)	3.79	4.431
30,56	3.85	4.617
63,64	1.81	1.766
15,30	3.06	1.849
30,(51,61,62)	4.86	4.956
30,57	2.69	3.685

RDC couplings (Hz)

Atoms	Experimental	Calculated Average
(44,58) (44,59) (44,60)	-18.88	-6.6
(23,51) (23,61) (23,62)	-13.11	-24.762
14,56	31.68	35.885
37,65	-71.27	-118.066
35,57	-91.15	-70.484
12,15	20.94	27.827
25,30	73.78	46.356
40,53	-2.66	7.614
42,55	29.4	25.868
41,54	66.79	96.954
4,21	57.09	34.581
6,18	54.77	47.837
39,52	26.32	28.988
5,19	36.29	17.867
3,20	46.76	48.052

Chemical shifts (ppm)

Atoms	Experimental	Calculated Average
35	87.1	87.074
57	5.48	5.101
28	87.8	86.937
32	133.3	134.816
39	126.8	126.957
52	7.78	7.584
40	127.5	124.719
53	7.37	6.918
41	132.8	131.908
54	7.56	7.105
42	118.4	113.821
55	7.53	7.04
33	142.2	139.085
26	172.5	173.712
25	56.9	56.5

30	5.93	5.702
29	32.6	37.959
63,64	3.16	2.804
36	179.2	174.967
37	61.6	67.063
65	3.83	3.525
10	163.1	161.708
2	121.8	120.534
3	127.5	126.526
20	8.24	7.814
4	129.2	127.482
21	7.6	7.169
5	136.5	135.382
19	7.89	7.444
6	128.5	127.369
18	7.76	7.219
1	147.7	147.126
8	154.8	155.782
12	81	77.523
15	5.64	4.872
14	33.5	37.119
56	2.49	2.449
16,17	19.3	17.526
45,46,47,48,49,50	1.07	0.752
22	171.8	175.239
23	20.7	20.015
51,61,62	2.22	1.774
44	17.9	16.317
58,59,60	1.5	1.247

Solution 5 (1 conformers), χ^2: 3043.316, AIC: 3043.316, Relative Probability: 0.000

Conformer 2: 100%

NOE distances (Å)

Atoms	Experimental	Calculated Average
57,(58,59,60)	4.12	3.1

15,(51,61,62)	3.79	4.428
30,56	3.85	2.053
63,64	1.81	1.767
15,30	3.06	3.562
30,(51,61,62)	4.86	4.081
30,57	2.69	3.627

RDC couplings (Hz)

Atoms	Experimental	Calculated Average
(44,58) (44,59) (44,60)	-18.88	-1.547
(23,51) (23,61) (23,62)	-13.11	-16.168
14,56	31.68	21.629
37,65	-71.27	-105.406
35,57	-91.15	-71.462
12,15	20.94	19.589
25,30	73.78	33.432
40,53	-2.66	9.487
42,55	29.4	48.694
41,54	66.79	77.44
4,21	57.09	25.104
6,18	54.77	57.135
39,52	26.32	50.086
5,19	36.29	25.243
3,20	46.76	57.012

Chemical shifts (ppm)

Atoms	Experimental	Calculated Average
35	87.1	86.94
57	5.48	5.08
28	87.8	87.003
32	133.3	134.481
39	126.8	126.924
52	7.78	7.57
40	127.5	124.657
53	7.37	6.896
41	132.8	132.031

54	7.56	7.126
42	118.4	113.8
55	7.53	7.029
33	142.2	139.118
26	172.5	173.696
25	56.9	57.14
30	5.93	5.017
29	32.6	37.051
63,64	3.16	2.741
36	179.2	174.743
37	61.6	67.104
65	3.83	3.5
10	163.1	162.341
2	121.8	120.278
3	127.5	126.415
20	8.24	7.834
4	129.2	127.772
21	7.6	7.165
5	136.5	135.5
19	7.89	7.454
6	128.5	127.73
18	7.76	7.209
1	147.7	146.836
8	154.8	153.393
12	81	83.859
15	5.64	5.33
14	33.5	36.716
56	2.49	2.009
16,17	19.3	18.184
45,46,47,48,49,50	1.07	0.804
22	171.8	173.737
23	20.7	20.324
51,61,62	2.22	1.913
44	17.9	16.294

58,59,60 1.5 1.239

Solution 6 (1 conformers), χ^2: 3979.427, AIC: 3979.427, Relative Probability: 0.000

Conformer 3: 100%

NOE distances (Å)

Atoms Experimental Calculated Average

57,(58,59,60) 4.12 3.821

15,(51,61,62) 3.79 4.441

30,56 3.85 4.589

63,64 1.81 1.775

15,30 3.06 1.94

30,(51,61,62) 4.86 4.318

30,57 2.69 3.297

RDC couplings (Hz)

Atoms Experimental Calculated Average

(44,58) (44,59) (44,60) -18.88 -8.885

(23,51) (23,61) (23,62) -13.11 14.934

14,56 31.68 37.577

37,65 -71.27 -78.274

35,57 -91.15 -62.685

12,15 20.94 38.332

25,30 73.78 33.738

40,53 -2.66 19.109

42,55 29.4 30.247

41,54 66.79 57.705

4,21 57.09 27.24

6,18 54.77 50.304

39,52 26.32 30.266

5,19 36.29 41.925

3,20 46.76 51.189

Chemical shifts (ppm)

Atoms Experimental Calculated Average

35 87.1 86.961

57 5.48 5.237

28 87.8 88.125

32	133.3	132.544	
39	126.8	125.715	
52	7.78	7.177	
40	127.5	125.226	
53	7.37	6.944	
41	132.8	131.658	
54	7.56	7.156	
42	118.4	115.898	
55	7.53	7.082	
33	142.2	140.984	
26	172.5	173.413	
25	56.9	57.399	
30	5.93	4.674	
29	32.6	34.141	
63,64		3.16	2.692
36	179.2	174.238	
37	61.6	65.044	
65	3.83	3.682	
10	163.1	162.108	
2	121.8	120.852	
3	127.5	126.35	
20	8.24	7.758	
4	129.2	127.734	
21	7.6	7.164	
5	136.5	135.177	
19	7.89	7.448	
6	128.5	127.633	
18	7.76	7.237	
1	147.7	146.736	
8	154.8	153.422	
12	81	77.05	
15	5.64	5.215	
14	33.5	34.646	
56	2.49	2.644	

16,17	19.3	17.237
45,46,47,48,49,50	1.07	0.736
22	171.8	175.233
23	20.7	20.328
51,61,62	2.22	1.809
44	17.9	19.257
58,59,60	1.5	1.279
