# Supplementary material for: Absolute Configuration of 12S-Deoxynortryptoquivaline from Ascidian-Derived Fungus Aspergillus clavatus Determined by Anisotropic NMR and Chiroptical Spectroscopy
Source: J Nat Prod. 2024 Jan 30;87(2):381–7. doi: 10.1021/acs.jnatprod.3c01157 (PMC10897928; doi:10.1021/acs.jnatprod.3c01157)
Supplement: Supplementary file 2 — np3c01157_si_002.zip [file np3c01157_si_002.zip › AS107_NMR/anisotropic_condition/Experiments and parameters.docx]

1. **File 1:** the 1D ^1^H NMR spectrum under initial anisotropic condition was acquired with following parameters: size of fid (32K), dummy scans (4), number of scans (4), and spectral width (16 ppm).
2. **File 2:** the 1D ^2^H NMR spectrum under initial anisotropic condition was acquired with following parameters: size of fid (32K), dummy scans (0), number of scans (1), and spectral width (100 ppm).
3. **File 3:** The 1D ^13^C NMR spectrum under initial anisotropic condition was acquired with following parameters: size of fid (128K), dummy scans (8), number of scans (8000), and spectral width (180 ppm).
4. **File 4:** the 1D ^1^H NMR spectrum under equilibrated anisotropic condition was acquired with following parameters: size of fid (32K), dummy scans (4), number of scans (4), and spectral width (16 ppm).
5. **File 11:** the 1D ^2^H NMR spectrum under equilibrated anisotropic condition was acquired with following parameters: size of fid (32K), dummy scans (0), number of scans (1), and spectral width (100 ppm).
6. **File 12:** the 1D ^2^H NMR spectrum under equilibrated anisotropic condition was acquired with following parameters: size of fid (32K), dummy scans (0), number of scans (1), and spectral width (100 ppm).
7. **File 13:** The 1D ^13^C NMR spectrum under equilibrated anisotropic condition was acquired with following parameters: size of fid (128K), dummy scans (8), number of scans (8000), and spectral width (180 ppm).
8. **File 14:** The 2D ^1^H-^13^C CLIP-HSQC NMR spectrum under equilibrated anisotropic condition was acquired with following parameters: size of fid (4 K in F2 dimension, 700 in F1 dimension), dummy scans (8), number of scans (26), spectral width (9 ppm in F2 dimension and 140 ppm in F1 dimension), and dwell time (74 *µ*sec). The experimental RDC data please see in table S2 in SI.
