# Supplementary material for: Absolute Configuration of 12S-Deoxynortryptoquivaline from Ascidian-Derived Fungus Aspergillus clavatus Determined by Anisotropic NMR and Chiroptical Spectroscopy
Source: J Nat Prod. 2024 Jan 30;87(2):381–7. doi: 10.1021/acs.jnatprod.3c01157 (PMC10897928; doi:10.1021/acs.jnatprod.3c01157)
Supplement: Supplementary file 2 — np3c01157_si_002.zip [file np3c01157_si_002.zip › AS107_NMR/isotropic_condition/Experiments and parameters.docx]

1. **File 1**: the 1D ^1^H NMR spectrum under isotropic condition was acquired with following parameters: size of fid (32K), dummy scans (4), number of scans (16), and spectral width (9 ppm). The acquired ^1^H data as following table using peaks of MeOH-*d*_4_ solvent as reference (3.31ppm for ^1^H nucleus):

| ***δ***_H_ **(*J* in Hz)** |
| --- |
| 5.48, s |
| 7.78, d (8.2) |
| 7.37, t (7.6) |
| 7.55, m |
| 7.52, m |
| 5.93, t (9.8) |
| 2.77, dd (13.1, 9.4) |
| 3.54, dd (13.1, 10.2) |
| 3.83, q (7.0) |
| 8.23, d (8.0) |
| 7.60, t (7.7) |
| 7.89, t (7.7) |
| 7.76, d (8.5) |
| 5.64, d (9.7) |
| 2.49, m |
| 0.97, d (6.5) |
| 1.17, d (6.7) |
| 2.22, s |
| 1.50, d (7.0) |

1. **File 2**: 1D ^13^C NMR spectrum under isotropic condition was acquired with following parameters: size of fid (128K), dummy scans (8), number of scans (9600), and spectral width (180 ppm). The acquired ^13^C data as following table using peaks of MeOH-*d*_4_ solvent as reference (49.0 ppm for ^13^C nucleus):

| ***δ*_C_, type** |
| --- |
| 87.1, CH |
| 87.8, C |
| 133.3, C |
| 126.8, CH |
| 127.5, CH |
| 132.8, CH |
| 118.4, CH |
| 142.2, C |
| 172.5, C |
| 56.9, CH |
| 32.6, CH_2_ |
| 179.2, C |
| 61.6, CH |
| 163.1, C |
| 121.8, C |
| 127.5, CH |
| 129.2, CH |
| 136.5, CH |
| 128.5, CH |
| 147.7, C |
| 154.8, C |
| 81.0, CH |
| 33.5, CH |
| 19.1, CH_3_ |
| 19.4, CH_3_ |
| 171.8, C |
| 20.7, CH_3_ |
| 17.9, CH_3_ |

1. **File 3**: the 2D ^1^H-^13^C CLIP-HSQC NMR spectrum under isotropic condition was acquired with following parameters: size of fid (4 K in F2 dimension, 700 in F1 dimension), dummy scans (8), number of scans (26), spectral width (9 ppm in F2 dimension and 140 ppm in F1 dimension), and dwell time (74 *µ*sec). The experimental RDC data please see in table S2 in SI.
2. **File 4**: the 2D ^1^H-^13^C HSQC NMR spectrum under isotropic condition was acquired with following parameters: size of fid (2 K in F2 dimension, 512 in F1 dimension), dummy scans (16), number of scans (22), spectral width (9 ppm in F2 dimension and 110 ppm in F1 dimension), and dwell time (74 *µ*sec).
3. **File 5:** the 2D ^1^H-^13^C HMBC NMR spectrum under isotropic condition was acquired with following parameters: size of fid (4 K in F2 dimension, 512 in F1 dimension), dummy scans (16), number of scans (30), spectral width (9 ppm in F2 dimension and 190 ppm in F1 dimension), and dwell time (74 *µ*sec).
4. **File 6:** the 2D ^1^H-^1^H COSY NMR spectrum under isotropic condition was acquired with following parameters: size of fid (4 K in F2 dimension, 1 K in F1 dimension), dummy scans (8), number of scans (16), spectral width (9 ppm in both F2 in F1 dimensions).
5. **File 7:** the 2D ^1^H-^1^H NOESY NMR spectrum under isotropic condition was acquired with following parameters: size of fid (4 K in F2 dimension, 1 K in F1 dimension), dummy scans (8), number of scans (24), spectral width (9 ppm in both F2 and F1 dimensions), and mixing time (350 msec). The acquired NOE peaks please see table S1 in SI.
